# Functional infrared thermography imaging can be used to assess the effectiveness of Maxicam Gel^®^ in pre-emptively treating transient synovitis and lameness in horses

**DOI:** 10.3389/fvets.2024.1399815

**Published:** 2024-06-11

**Authors:** Júlia Ribeiro Garcia de Carvalho, Debora Del Puppo, Thayssa de Oliveira Littiere, Nathali Adrielli Agassi de Sales, Ana Carolina Yamamoto Silva, Gesiane Ribeiro, Ferdinando Nielsen de Almeida, Bruna Gomes Alves, Igor Renan Honorato Gatto, Gabriel Vieira Ramos, Guilherme de Camargo Ferraz

**Affiliations:** ^1^Laboratory of Equine Exercise Physiology and Pharmacology (LAFEQ), Department of Animal Morphology and Physiology, School of Agricultural and Veterinary Sciences, São Paulo State University, FCAV/UNESP, São Paulo, Brazil; ^2^Research and Development Department, Ourofino Animal Health Company, São Paulo, Brazil; ^3^Veterinary and Animal Research Centre (CECAV), Faculty of Veterinary Medicine, Lusófona University - Lisbon University Centre, Lisbon, Portugal; ^4^Equine Sports Medicine Laboratory, Department of Veterinary Medicine and Surgery, School of Agricultural and Veterinary Sciences, São Paulo State University, FCAV/UNESP, São Paulo, Brazil

**Keywords:** analgesic, lameness, lipopolysaccharide, meloxicam, nonsteroidal anti-inflammatory drug, soreness, cyclooxygenase, osteoarthritis

## Abstract

**Introduction:**

Diagnosing and treating lameness in horses is essential to improving their welfare. In equine orthopedic practice, infrared thermography (IRT) can indirectly detect soreness. Non-steroidal anti-inflammatory drugs can treat painful and inflammatory processes in horses. Using IRT, the efficacy of meloxicam (Maxicam Gel^®^) was evaluated in pre-treating transient synovitis in horses induced by a middle carpal joint injection of lipopolysaccharides (LPS) from *E. coli* 055:B5 at a dose of 10 endotoxin units.

**Methods:**

In a cross-over design, six healthy horses were randomly assigned to receive either 0.6 mg/kg of oral Maxicam Gel^®^ (MAXVO) or a mock administration (control group, C) following a two-week washout period. IRT of the middle carpal joint, visual lameness assessment and joint circumference were recorded over time. Clinical and hematological evaluations were performed. Synovial fluid aspirates were analyzed for total nucleated cell count, total protein, and prostaglandin E_2_. A mixed effects analysis of variance was performed for repeated measures over time, followed by Tukey’s test. A multinomial logistic regression was conducted to determine whether there is a relationship between a thermography temperature change and the lameness score.

**Results:**

There were no changes in joint circumference. The MAXVO group showed a lower rectal temperature 4 h after synovitis induction. The C group presented an increase in neutrophils and a decrease in total hemoglobin and hematocrit 8 h after induction. No changes were observed in the synovial fluid between groups. The horses that received meloxicam did not show clinically significant lameness at any time, while the C group showed an increase in lameness 2, 4, and 8 h after synovitis induction.

**Discussion:**

IRT indicated that the skin surface temperature of the middle carpal joint was lower in horses who received meloxicam, suggesting a reduction in the inflammatory process induced by LPS. It was observed that the maximum temperature peaks in the dorsopalmar and lateropalmar positions can be utilized to predict the severity of lameness, particularly when the temperature rises above 34°C. Horses pre-treated with meloxicam showed either reduced or no indication of mild to moderate pain and presented a lowehr thermographic temperature, which indicates the effectiveness of Maxicam Gel^®^ as an anti-inflammatory.

## 1 Introduction

Diagnosing and treating lameness in horses is crucial for ensuring their welfare in the equine field practice. One helpful approach to diagnosis is thermal imaging, which estimates the pathophysiological response to a noxious stimulus through heat production (1). To manage inflammatory and painful conditions in horses, non-steroidal anti-inflammatory drugs (NSAIDs) are commonly administered to treat musculoskeletal diseases (2). These drugs are potent inhibitors of cyclooxygenase enzymes (COXs), which are responsible for converting arachidonic acid into eicosanoids such as prostaglandins, leukotrienes, and thromboxanes. These chemical mediators play an essential role in the inflammation cascade, including increased vascular permeability and heat. Blocking the production of these mediators causes an anti-inflammatory and analgesic effect (3, 4).

COX-1 is constitutively expressed in almost all body tissues, acting in routine and physiological functions, including gastrointestinal homeostasis, through cytoprotection of the gastric epithelium, and maintenance of renal blood perfusion. COX-2 is an enzyme up-regulated by cytokines that show increased activity during pro-inflammatory states. Unlike COX-1, COX-2 has relatively low activity under physiological conditions (3, 5, 6). In recent decades, drugs with greater selectivity for COX-2 have been developed, such as meloxicam, an NSAID belonging to the enolic acid class. The main advantage of COX-2 selective NSAIDs is the reduction in adverse effects associated with the gastrointestinal tract, such as gastric ulcerations and colitis (6). In rats, ulcerogenicity in the stomach is weak compared to anti-inflammatory potency, resulting in a high therapeutic index (7).

Meloxicam effectively relieves postoperative pain in human patients undergoing several types of surgeries, such as knee osteoarthritis surgery. For instance, a trail showed that two oral doses preemptively administered of meloxicam improved postoperative pain control in patients receiving arthroscopic knee surgery (8). According to these authors, meloxicam’s potential as a preemptive analgesic in postoperative pain control is preliminary but promising. In equine medicine, more information on the preemptive use of meloxicam that can be applied clinically needs to be present in the literature.

Some studies have shown that meloxicam is effective in treating lameness in horses caused by synovitis induced by intra-articular administration of lipopolysaccharides (LPS), principally model of inflammatory pain. These studies have used various methods and biomarkers to test the clinical effectiveness of meloxicam. The administration of meloxicam has been found to reduce clinical lameness scores (5, 9–11), mitigate the asymmetry of head movement (5), and suppress inflammatory markers such as total protein (TP), total nucleated cell count (TNCC), and prostaglandin E_2_ (PGE_2_) in synovial fluid (11, 12). Thus, there is scientific evidence to suggest that meloxicam can reduce musculoskeletal pain of inflammatory origin in horses.

Another technique that demonstrates potential for detecting NSAIDs’ effectiveness is infrared thermography (IRT), a noninvasive, radiation free, practical, fast, and low-cost method. Evaluation of the surface temperature can be used as a diagnostic tool to accurately estimate the thermal, metabolic, and vascular conditions of the equine body. Changes in local perfusion, such as vasodilation, can cause alterations in the body surface temperature. Increases in vascularization and blood supply to tissues are the basis for thermography diagnosis. Temperature is a crucial physical property that can directly reflect joint inflammation. Thus, IRT can be used to map classic inflammatory clinical signs such as heat, which indicates changes in skin surface temperature caused by vascular or inflammatory alterations (1, 13–15).

Heat is one of the key signs of inflammation, as the increased blood supply caused by inflammation leads to an increase in local temperature (16). IRT has been used in various studies involving horses, such as detection of jugular venipuncture for anti-doping control in equestrian events (17), monitoring of musculoskeletal adaptation to training (18), evaluation of the welfare in athlete horses (19), monitoring the effects of training (20, 21), assessment of thermoregulation during exercise (22), validation for the use of eye temperature as an indicator of well-being (23), detection of pregnancy in mares (24), evaluation of the oral administration of meloxicam or flunixin meglumine in an inflammatory response induced by the administration of systemic LPS (25), evaluation of thermoregulation using two methods of post-exercise cooling (26), evaluation of biocompatibility of polymers (27, 28), among others.

IRT has been used in humans to screen soft tissue and measure joint skin temperature to diagnose conditions such as rheumatoid arthritis, osteoarthritis, and ankle sprains (15, 29–31). In bovines, IRT was used to compare the effectiveness of oral meloxicam and intravenous flunixin meglumine in controlling lameness-associated pain in lactating dairy cattle (32). This technique was also used to evaluate dogs with hip osteoarthritis (33).

More research is still needed on the use of IRT to indirectly assess pain and inflammation in domestic animals (1). However, IRT is a currently available method to detect soreness in horses (34). When it comes to using IRT to prove the effectiveness of NSAIDs, there is a relative scarcity of studies in horses. Some studies have used IRT to assess the vascular component of the inflammatory response and examine the effects of anti-inflammatory drugs on experimentally induced acute inflammation (35, 36). Recent studies have induced systemic inflammation by administering LPS intravenously and used IRT to evaluate the effectiveness of NSAIDs by analyzing the temperature of the hoof wall of horses (25, 37). Furthermore, IRT has been shown to be effective in detecting intrasynovial injections in horses (38).

The purpose of this study was to investigate the effectiveness of non-steroidal anti-inflammatory drugs (NSAIDs) using IRT as a non-invasive diagnostic method. The authors report on the use of IRT to quantify the degree of inflammation in horses with experimentally induced transient synovitis with LPS. These horses had previously received meloxicam, an older COX-2 selective inhibitor.

## 2 Materials and methods

### 2.1 Ethics statement

The study adhered to the Ethical Principles in Animal Experimentation as established by the National Council for Control in Animal Experimentation (CONCEA). The protocol underwent review and approval by the Ethics Committee on the Use of Animals—CEUA—UNESP, Jaboticabal, Brazil (Protocol No. 2887/2021).

### 2.2 Horses

Six crossbreed horses, three males and three females, were used for the experiment. They weighed an average of 395 ± 35 kg and aged between 12 and 20 years. These horses belong to the didactic herd of the Equine Exercise Physiology and Pharmacology Laboratory (LAFEQ), Department of Animal Morphology and Physiology, School of Agricultural and Veterinarian Sciences, São Paulo State University (FCAV/UNESP), Jaboticabal, São Paulo, Brazil. They were kept in a paddock and fed with 0.05% of body weight in concentrate once a day, along with hay, hay silage, mineral salt, and water *ad libitum*. Before the experiment began, the horses underwent a complete physical examination to ensure their health status. Haematological and biochemical tests were also conducted. The horses were previously treated with anthelmintics and vaccinated against rabies, tetanus toxoid, eastern and western equine encephalomyelitis, and equine influenza types A1 and A2.

### 2.3 Experimental groups

Based on a former study (11), the study’s sample size was six horses, all housed under the same condition. The horses were distributed into two groups: the control group (C, *n* = 6) and the group treated with meloxicam (MAXVO, *n* = 6). The meloxicam was formulated and manufactured by Ourofino Animal Health Company for commercial use in Brazil, and the study was designed to meet Brazilian regulations. The study was conducted in a controlled cross-over design in a paired, blinded, randomised experiment, with a two-week washout between phases. The MAXVO group was given meloxicam orally at a dose of 0.6 mg/kg of body mass, once per day for 3 days, at 48 h (D-2), 24 h (D-1), and 1 h (0) before synovitis induction, according to a previously adapted protocol (11). If the horses still showed lameness, an additional dose was given 24 h after synovitis induction. The horses in the control group received a saline solution orally at the exact times as the MAXVO group to simulate the same conditions as the meloxicam administration. As mentioned, the study was conducted blindly, and the researchers responsible for evaluations were not aware of which group each horse belonged to. Only one researcher (JC) was responsible for administering treatments to the horses.

### 2.4 Preparation of LPS

Standardization of laboratory procedures for preparing the LPS solution is crucial. A previously established LPS model, described in Standardbred, was followed (11). The solution of *E. coli* 055:B5 (L2880, Sigma Aldrich, lot 059N4031V) was prepared using sterile materials, and all stages of solution preparation were carried out in a laminar flow, under refrigeration. From the stock solution, which had a concentration of 5 mg/mL of LPS in RPMI 1640 Medium (Gibco^TM^, Thermo Fisher), a new dilution was prepared by adding 1 μL of the stock solution to 5 mL of RPMI. This intermediate solution had a concentration of 3,000 EU/mL and was stored in an appropriate flask. The working solution was obtained by diluting 90 μL of the intermediate solution in 27 mL of sterile PBS, resulting in a desired concentration of 10 EU/mL. The working solution was stored in microtubes containing 1.5 mL each, and the material was thawed for a maximum of 1 h before intra-articular injection to induce the inflammatory process. The stock and intermediate solutions were vortexed for 10 min at a speed of 1,500 rpm before dilution to ensure a homogeneous solution (11). Before intra-articular administration, the working solution was vortexed again for 2 min at 1,500 rpm.

### 2.5 Induction of the inflammatory process

The horses underwent a procedure that induced a temporary inflammatory process by applying *E. coli* 055:B5 LPS to their middle carpal joint. A random drawing determined which joint (left or right) would receive the 1 mL solution containing 10 endotoxin units (EU) of LPS. To ensure the safety of the procedure, the horses were sedated with detomidine (0.01 mg/kg i.v.) and then given yohimbine (0.12 mg/kg i.v.) to reverse the sedation after the procedure was completed.

The initiation of inflammation was designated as the baseline time point (0). Before the arthrocentesis procedure, the application site was cleaned with chlorhexidine degermant and 70% alcohol to ensure antisepsis. A 30 × 8 needle and a 1 mL syringe were used to collect the synovial fluid. After that, the entire LPS solution was injected into both groups. Following the induction of transitory synovitis, the horses were kept in individual paddocks with an area of approximately 75.9 m^2^. These paddocks were surrounded by a wooden fence and smooth wire protected with plastic insulation and had a roof to protect against precipitation and intense sunlight. This type of management ensured the maintenance of regular habits of the equine species, such as grazing, moving, and socializing.

### 2.6 Assessment methods

#### 2.6.1 Clinical and hematological evaluation

Throughout the experiment the horses were being closely monitored by veterinarians to detect any signs of systemic clinical issues that might arise due to the administration of LPS. The horses were evaluated clinically by a physical examination, and blood samples were collected for hematological analysis (complete blood count). Heart rate (HR), respiratory rate (RR), rectal temperature (RT), degree of hydration, the color of the apparent mucous membranes (1 = pink, 2 = light pink, 3 = reddish, 4 = white, 5 = yellowish, 6 = bluish), behavior (0 = normal, 1 = apathetic; 2 = restless), and appetite (0 = normal, 1 = hyporexia; 2 = anorexia) were all measured. The horses were evaluated at several time points: five days (D-5), two days (D-2), one day (D-1), and immediately before the induction of the inflammatory process, and 0, 2, 4, 6, 8, 12, 24, and 48 h after synovitis induction with LPS. Blood samples were collected by venipuncture of the jugular vein in negative pressure tubes containing ethylenediaminetetraacetic acid (EDTA). The samples were kept in isothermal boxes with recyclable ice and transported to the laboratory. A hematological analyzer (ABX Micros 60, Horiba) was used. Blood collection was performed at the following time points: 0, 8, 24, and 48 h after the induction of the inflammatory process.

#### 2.6.2 Synovial fluid

Synovial fluid was obtained via aseptic arthrocentesis of the middle carpal joint. The animals were chemically restrained using detomidine (0.01 mg/kg i.v.) and the application site was made aseptic using chlorhexidine degermant and 70% alcohol. Synovial fluid was collected using a 30 × 8 needle. Samples were divided into 0.5 mL pediatric tubes containing EDTA and plain microtubes and were immediately stored at 4°C. Samples in EDTA tubes were analyzed, whereas plain tubes were processed within 1 h after collection. Synovial fluid was collected at 0, 8, 24, and 48 h after induction of the inflammatory process. The animals were chemically reverted from sedation using yohimbine (0.12 mg/kg i.v.). Aliquots of synovial fluid collected in tubes with EDTA were used to perform TNCC and TP assays. The plain tubes were centrifuged at 2,000 G for 20 min at 4°C. The supernatant was then collected and distributed into 500 μL aliquots, placed in microtubes without anticoagulant, and stored at −80°C until the moment of quantification of PGE_2_. The quantification of PGE_2_ was performed using the Prostaglandin E_2_ EIA Kit (Elabscience, Texas, United States) through ELISA. The intra-assay coefficient of variation for PGE_2_ was 1.2%.

#### 2.6.3 Joint circumference

To assess inflammation and oedema, joint circumference was measured using a measuring tape placed immediately distal to the accessory carpus. The measurements were taken at the following time points: 0, 2, 4, 6, 8, 12, 24, and 48 h after the inflammation was induced.

#### 2.6.4 Lameness

During the study, the horses were evaluated to detect movement asymmetries for their degree of lameness. Visual examination was done by inspecting the horses while they trotted in a straight line over a hard surface for approximately 40 meters. The evaluation was conducted by an experienced clinician (GCF) unaware of the experimental groups. The clinician assigned scores to the horses based on the following criteria: 0 for absence of visible lameness, (1) for discrete asymmetry that was occasionally inconsistent, (2) for visible lameness that was rarely inconsistent, (3) for visible lameness always, and (4) for complete inability to bear weight (39). The evaluations were performed at 0, 2, 4, 8, 24, and 48 h after the induction of the inflammatory process.

#### 2.6.5 Infrared thermography

**Figure 1:**
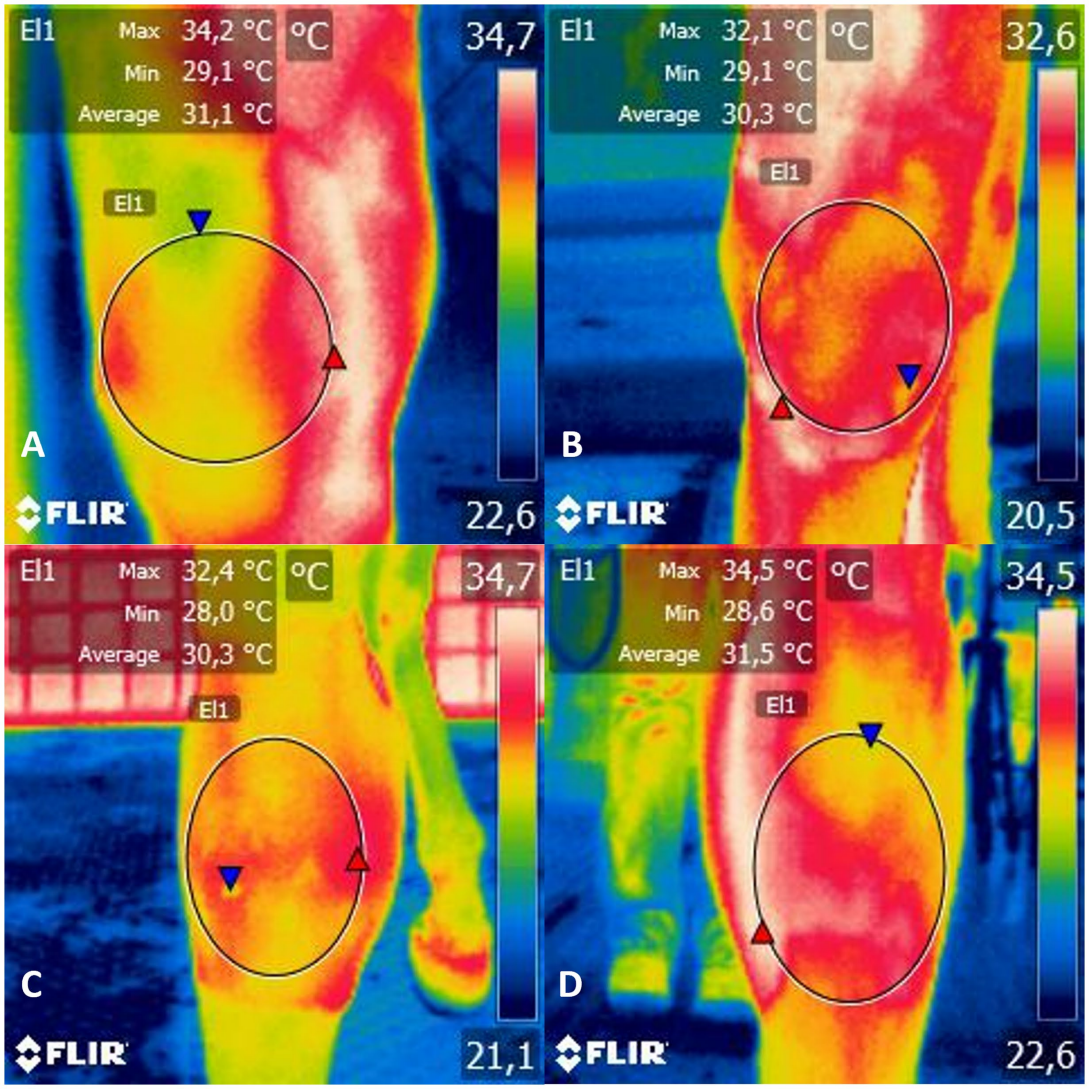
Infrared thermogram of the **(A)** mediolateral, **(B)** lateromedial, **(C)** dorsopalmar and **(D)** palmarodorsal view of the carpal joint of horses submitted to experimental induction of transient synovitis for both trials (C and MAXVO). C, control group; MAXVO received meloxicam (Maxicam Gel^®^), administered orally at 0.6 mg/kg daily 3 days before LPS injection. Observe temperature scale on the right. The circle delimits the evaluated area.

Functional IRT imaging is a useful diagnostic tool in veterinary medicine for examining inflammation. The proper use of thermography requires a controlled environment and adherence to imaging protocol to eliminate errors. Thermographic examinations were conducted on the joint that received LPS and the opposite joint, which was used as a negative control. Four images were taken for each limb of the carpal anatomic region: mediolateral (ML), lateromedial (LM), dorsopalmar (DP), and palmarodorsal (PD) views (Figure 1). The horses were led into a closed, airy environment that was free from drafts and direct sunlight (14). Before taking the images, the carpal anatomic region on both limbs was gently cleaned with dry gauze, and the area was not touched again after cleaning. These procedures were carried out to allow the horse to acclimate to the ambient environmental factors. The temperature and humidity of the environment were controlled to standardize the thermal measurements. The horses were not sedated during the evaluations. It is worth noting that before the examinations, the cutaneous area of the carpal joint was clipped at least 24 h before induction of synovitis to minimize errors caused by reflection or refraction.

An infrared thermographic camera from Flir^®^ Systems (model i50, Wilsonville, Oregon, United States) was used for the assessment. Based on the technical specifications provided by the manufacturer, the camera’s sensitivity value is less than 0.1°C, with an emissivity of 0.98, temperature range from −20 to 350°C, image frequency of 9 Hz, resolution of 140 × 140 pixels, and an accuracy of ±2%. The thermographic camera was placed perpendicular to the assessment site at a 0.4 m distance to capture infrared thermographic images. All thermographic images were taken by the same examiner (TOL). Image analysis was performed using FLIR Tools software from FLIR Systems Inc. (Wilsonville, OR, United States). An area was marked on the thermographic image across the entire carpal region. It should be noted that the middle carpal joint, where the LPS was injected, communicates with the small carpometacarpal joint situated between the third and fourth carpal bones (40). The software calculated the delimited area’s minimum, maximum, and average thermographic temperatures. The assessments were carried out at 0, 2, 4, 6, 8, 12, 24, and 48 h after the induction of the inflammatory process.

### 2.7 Statistical design and analysis

The data was analyzed using SigmaPlot software (v.12.5) and tested for normality using the Shapiro–Wilk test. The experimental design was a factorial design with three paired replications in a split-split-plot scheme. Each split-plot was a treatment (C and MAXVO) and each split-split-plot was an examination time point (eight-time points for thermographic examination). In the next phase of the experiment, a cross-over design was applied, where the three horses that received Maxicam Gel^®^ in the first phase became controls in the second phase and vice versa, resulting in a total of six animals per experimental group. The model $Y_{\text{ijkl}=\mu +\alpha_{i}+\beta_{ij}+tk+{\left(\alpha \beta \right)}_{ik}+\in_{ijk}}$ was used, in which *Υ_ijkl_* is observation at the meloxicam factor level I; *μ* is the overall mean common to all observations; *α* is the effect of treatment (meloxicam); *β* is the effect of time point (in hours); and the *αβ* = effect of the interaction between treatment *i* and time *k*. A mixed model was applied with the horse as a random effect and time point, treatment, and their interaction as categorical fixed effects. The treatment effect was also tested at different times separately, according to the time points of each variable for the studied variables after synovitis induction. A mixed effects analysis of variance was performed for repeated measures over time, and means were compared using Tukey’s test. For the degree of lameness, the Wilcoxon non-parametric test was used for the intergroup comparison. For the intragroup comparisons, the Kruskal–Wallis one-way analysis of variance on ranks was used, and the means were compared using the Tukey test. A multinomial logistic regression (MLR) classification algorithm was conducted with R Programming Language (version 2024.04.0 + 735 “Chocolate Cosmos” release for windows) using multinon function [“nnet” package (version 7.3–19)]. To evaluate the predictive potential of thermography variables, such as minimum peak, maximum peak, and average peak thermographic temperatures, and anatomic regions ML, LM, DP, and PD, the MLR was employed. The goal was to accurately classify and predict the lameness score based on these variables. This model was used given the classification of lameness into five scores (0 to 4, considered the outcome variables) and the ability of the MLR to evaluate outcome variables with more than two categories. Before applying the MLR, the independence of irrelevant alternatives (IIA), the independence of errors (IE) and the absence of multicollinearity were checked. The first two premises were evaluated through visual inspection of the data, ensuring two similar categories in the outcome variable (for IIA assumption) and the absence of clusters within the experimental group used (for IE assumption). The assessment of multicollinearity was evaluated using the variance inflation factor (VIF) using vif function [“car” package (version 3.1–2)] with a threshold of 5. Variables that presented VIF >5 had a high correlation with the other variables included in the model and were removed from the analysis. To create the graphs, the functions ggplot [“ggplot2” package (version 3.4.4)], ggeffect [“ggeffects” package (version 1.5.2)] and ggarrange [“ggpubr” package (version 0.6.0)] were used. A Monte Carlo simulation was performed to create hundred temperature samples using the normal distribution. The means and standard deviation of each treatment and time point were taken from the original data. The simulation was also adjusted for ±2% accuracy. We applied a paired *t*-test to compare the generated data points to better understand the dispersion of the temperature data. Overall significance was set at 5% (*p* < 0.05).

## 3 Results

### 3.1 Clinical and hematological evaluation

Clinical and hematological variables were measured to detect possible signs of systemic sepsis caused by the intra-articular administration of LPS. The C group showed a higher RT 4 h after synovitis induction. Upon intragroup comparison, it was observed that in both groups the RR initially increased and then returned to its initial values. Additionally, an increase in RT was noted, especially between 4 and 12 h after synovitis induction, for both groups (Table 1). It is worth noting that most of the average values of RR and RT remained within the reference range for the equine species (41). Furthermore, there were no changes observed in the degree of hydration, colouration of the apparent mucous membranes, behaviour, or appetite of the animals. Table 2 shows no changes in erythrocytes, leukocytes, and lymphocytes. Group C exhibited a decrease in hemoglobin and hematocrit 8 h after induction. Neutrophilia was observed only at the eight-hour mark for group C.

**Table 1:** Means ± standard deviation values of heart rate (HR), respiratory rate (RR), and rectal temperature (RT) values of horses submitted to experimental induction of transient synovitis for both trials (C and MAXVO).

Assessment times (days/hours)	HR (bpm)		RR (mpm)		RT (°C)	
	C	MAXVO	C	MAXVO	C	MAXVO
D-5	46 ± 9	42 ± 6	18 ± 8^ab^	15 ± 5^ab^	37.1 ± 0.5^a^	37.0 ± 0.3^ab^
D-2	49 ± 11	39 ± 7	18 ± 6^ab^	18 ± 8^ab^	37.0 ± 0.4^a^	37.0 ± 0.4^ab^
D-1	49 ± 8	48 ± 17	19 ± 4^ab^	17 ± 7^ab^	37.0 ± 0.6^a^	36.7 ± 0.5^ad^
0	44 ± 10	53 ± 16	15 ± 9^ab^	14 ± 6^ab^	37.2 ± 0.4^ab^	37.3 ± 0.3^bcd^
2	45 ± 8	51 ± 14	16 ± 7^ab^	21 ± 11^a^	37.4 ± 0.2^abc^	37.3 ± 0.3^bc^
4	46 ± 6	48 ± 11	20 ± 10^a^	19 ± 7^ab^	38.0 ± 0.4^cd^	37.5 ± 0.3^*bc^
6	49 ± 17	44 ± 12	19 ± 7^a^	15 ± 7^ab^	37.6 ± 0.7^ad^	37.6 ± 0.2^bc^
8	46 ± 13	48 ± 13	15 ± 7^ab^	11 ± 4^b^	37.9 ± 0.3^bd^	37.5 ± 0.2^bc^
12	42 ± 6	43 ± 10	12 ± 4^ab^	13 ± 5^ab^	37.8 ± 0.4^bd^	37.8 ± 0.3^c^
24	43 ± 9	40 ± 4	12 ± 6^ab^	14 ± 6^ab^	37.1 ± 0.3^a^	37.0 ± 0.2^ab^
48	36 ± 5	43 ± 12	10 ± 2^b^	13 ± 7^ab^	37.0 ± 0.3^a^	37.3 ± 0.4^abc^

C, control group; MAXVO received meloxicam (Maxicam Gel^®^), administered orally at 0.6 mg/kg daily 3 days before LPS injection. Synovitis induction by injection of the middle carpal joint with 10 EU LPS occurred at 0 h. Lowercase letters indicate intragroup differences over time. ^*^Indicates difference between experimental groups.

**Table 2:** Means ± standard deviation values of erythrocytes, hemoglobin, hematocrit, leukocytes, segmented neutrophils, and lymphocytes of horses submitted to experimental induction of transient synovitis for both trials (C and MAXVO).

Variable	Groups	Assessment times (hours)			
		0	8	24	48
Erythrocytes (×10^6^/μL)	C	6.83 ± 0.23	6.70 ± 1.39	6.52 ± 0.59	6.42 ± 0.52
	MAXVO	6.67 ± 0.59	6.23 ± 0.66	6.42 ± 0.57	6.43 ± 0.69
Hemoglobin (g/dL)	C	11.72 ± 0.52^a^	10.38 ± 0.85^b^	11.1 ± 0.73^ab^	11.22 ± 0.80^ab^
	MAXVO	11.28 ± 0.83	10.85 ± 1.05	11.13 ± 0.75	11.15 ± 0.69
Hematocrit (%)	C	34.83 ± 1.72^a^	31.50 ± 2.43^b^	33.50 ± 2.35^ab^	32.67 ± 2.73^ab^
	MAXVO	34.17 ± 2.32	32.33 ± 2.50	33.50 ± 2.07	33.33 ± 2.07
Leukocytes (μL)	C	12,550 ± 3,352	13,483 ± 2,221	11,050 ± 2,268	10,700 ± 908
	MAXVO	10,600 ± 2,261	12,133 ± 2,734	10,267 ± 2,998	10,367 ± 3,092
Segmented neutrophils (μL)	C	7,599 ± 1,640^a^	9,782 ± 1,632^b^	7,084 ± 1,602^a^	6,467 ± 725^a^
	MAXVO	6,050 ± 1,021	7,871 ± 2,008	6,524 ± 2,185	6,215 ± 1,970
Lymphocytes (μL)	C	4,069 ± 1,805	2,937 ± 934	3,159 ± 590	3,293 ± 493
	MAXVO	3,592 ± 1,343	3,110 ± 587	3,023 ± 748	3,265 ± 1,242

C, control group; MAXVO received meloxicam (Maxicam Gel^®^), administered orally at 0.6 mg/kg daily 3 days before LPS injection. Synovitis induction by injection of the middle carpal joint with 10 EU LPS occurred at 0 h. Lowercase letters indicate intragroup differences over time.

### 3.2 Synovial fluid

No significant differences in the synovial fluid variables were displayed between groups (Table 3). A rise in TNCC was observed 8 h post-injection in both groups but returned to baseline concentrations after 48 h. Similarly, TP concentrations were elevated in both trials 8 and 24 h after the induction of transient synovitis but decreased after 48 h. Over time, PGE_2_ concentrations increased 24 and 48 h in C, and 48 h in MAXVO after the induction of transient synovitis compared to baseline.

**Table 3:** Means ± standard deviation of synovial fluid total nucleated cell count (TNCC), total protein (TP), and prostaglandin E_2_ (PGE_2_) of horses submitted to experimental induction of transient synovitis for both trials (C and MAXVO).

Assessment times (hours)	TNCC (×10^3^/mm^3^)		TP (mg/dL)		PGE_2_ (pg/mL)	
	C	MAXVO	C	MAXVO	C	MAXVO
0	0.21 ± 0.15ª	0.25 ± 0.16ª	1.27 ± 0.38ª	1.18 ± 0.42ª	179 ± 116^a^	136 ± 25^a^
8	96.7 ± 58.69^b^	113.07 ± 77.66^b^	4.78 ± 0.78^c^	5.22 ± 1.07^c^	281 ± 296^ab^	188 ± 71^ab^
24	30.13 ± 21.27ª	13.25 ± 13.08ª	4.22 ± 0.73^c^	4.33 ± 0.64^c^	355 ± 306^b^	404 ± 524^ab^
48	0.82 ± 0.73^a^	0.57 ± 0.16^a^	2.83 ± 1.15^b^	2.73 ± 0.46^b^	367 ± 267^b^	339 ± 265^b^

C, control group; MAXVO received meloxicam (Maxicam Gel^®^), administered orally at 0.6 mg/kg daily 3 days before LPS injection. Synovitis induction by injection of the middle carpal joint with 10 EU LPS occurred at 0 h. Lowercase letters indicate intragroup differences over time.

### 3.3 Joint circumference

**Figure 2:**
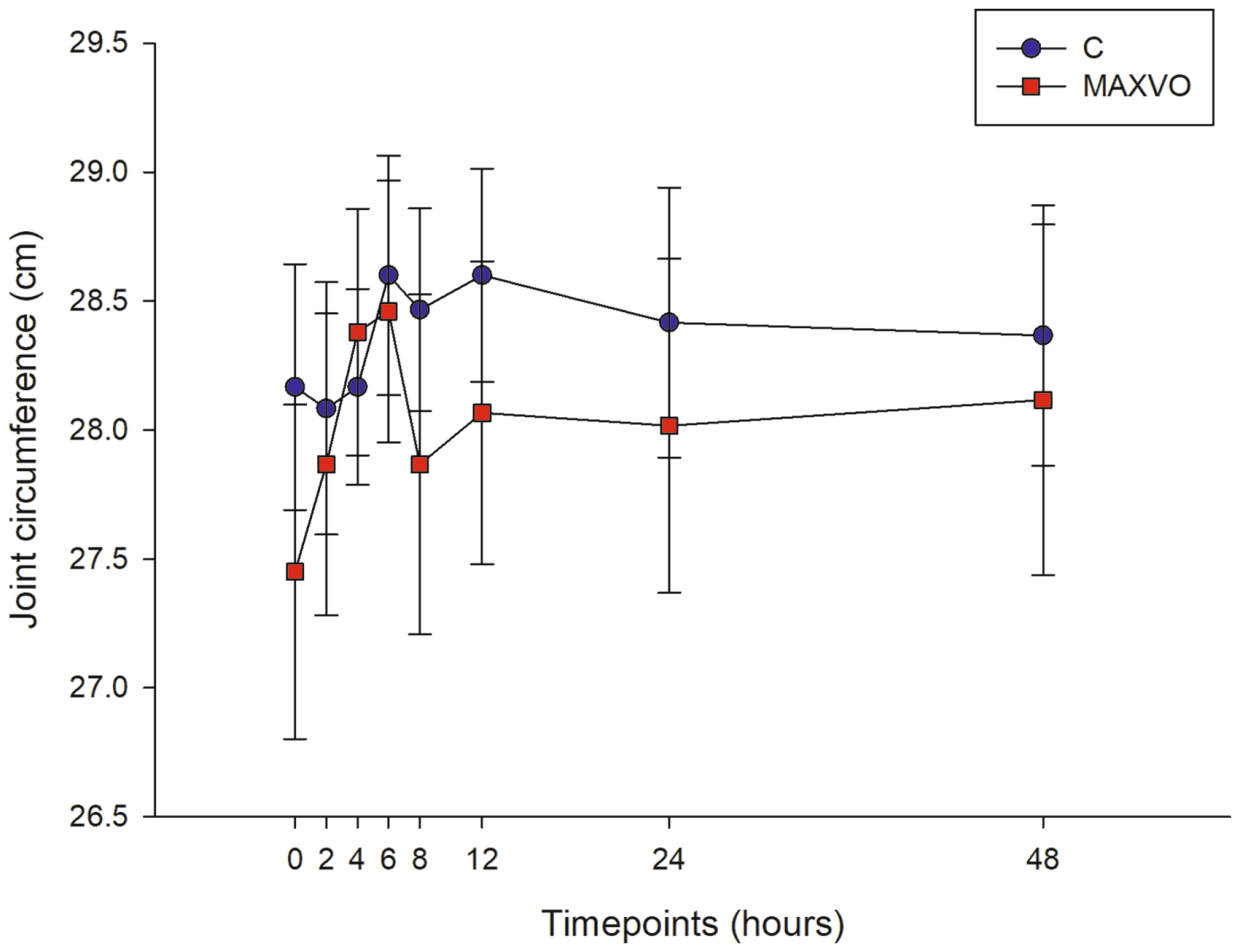
Graphical representation of means ± standard error of joint circumference of horses submitted to experimental induction of transient synovitis for both trials (C and MAXVO). C, control group; MAXVO received meloxicam (Maxicam Gel^®^), administered orally at 0.6 mg/kg daily 3 days before LPS injection. Synovitis induction by injection of the middle carpal joint with 10 EU LPS occurred at 0 h.

During the evaluation period, joint circumference was measured to indirectly assess joint swelling, which is indicated by effusion and periarticular edema. No statistically significant differences in joint circumference were observed within or between groups (Figure 2).

### 3.4 Lameness

Table 4 presents the median lameness scores 0, 2, 4, 8, 24, and 48 h after synovitis induction. A difference was observed for group C at 2, 4, and 8 h after synovitis induction, with the peak of lameness at 4 h. The median clinical lameness score was 3 (with a range of 3–3) in the C group, while it was 1 (with a range of 0–2) in the MAXVO group. The group that received meloxicam showed a reduction in lameness at all time intervals (2, 4, 8, 24, and 48 h) after synovitis induction (Supplementary Figure S1). As stated in the material and methods under item 2.3, if the horses continued to show signs of lameness, they would receive an additional dose of treatment 24 h after the LPS injection. Only one horse from the MAXVO group and three horses from the control (placebo) group received this additional dose.

**Table 4:** Medians (interquartile range) of the degree of clinical lameness of horses submitted to experimental induction of transient synovitis for both trials (C and MAXVO).

Assessment times (hours)	Degree of lameness		*p*
	C	MAXVO	
0	0 (0–0)^a^	0 (0–0)^a^	NS
2	3 (2–3)^b^	0 (0–0.25)^a*^	0.003
4	3 (3–3)^b^	1 (0–2)^a*^	0.002
8	3 (1.75–3)^b^	1 (0–1.25)^a*^	0.016
24	1.5 (0.75–2)^ab^	0 (0–0.25)^a*^	0.024
48	1 (1–1)^ab^	0 (0–0)^a*^	0.001

C, control group; MAXVO received meloxicam (Maxicam Gel^®^), administered orally at 0.6 mg/kg daily 3 days before LPS injection. Synovitis induction by injection of the middle carpal joint with 10 EU LPS occurred at 0 h. Lowercase letters indicate intragroup differences over time. ^*^Indicates difference between experimental groups. NS, not significant.

### 3.5 Infrared thermography

**Figure 3:**
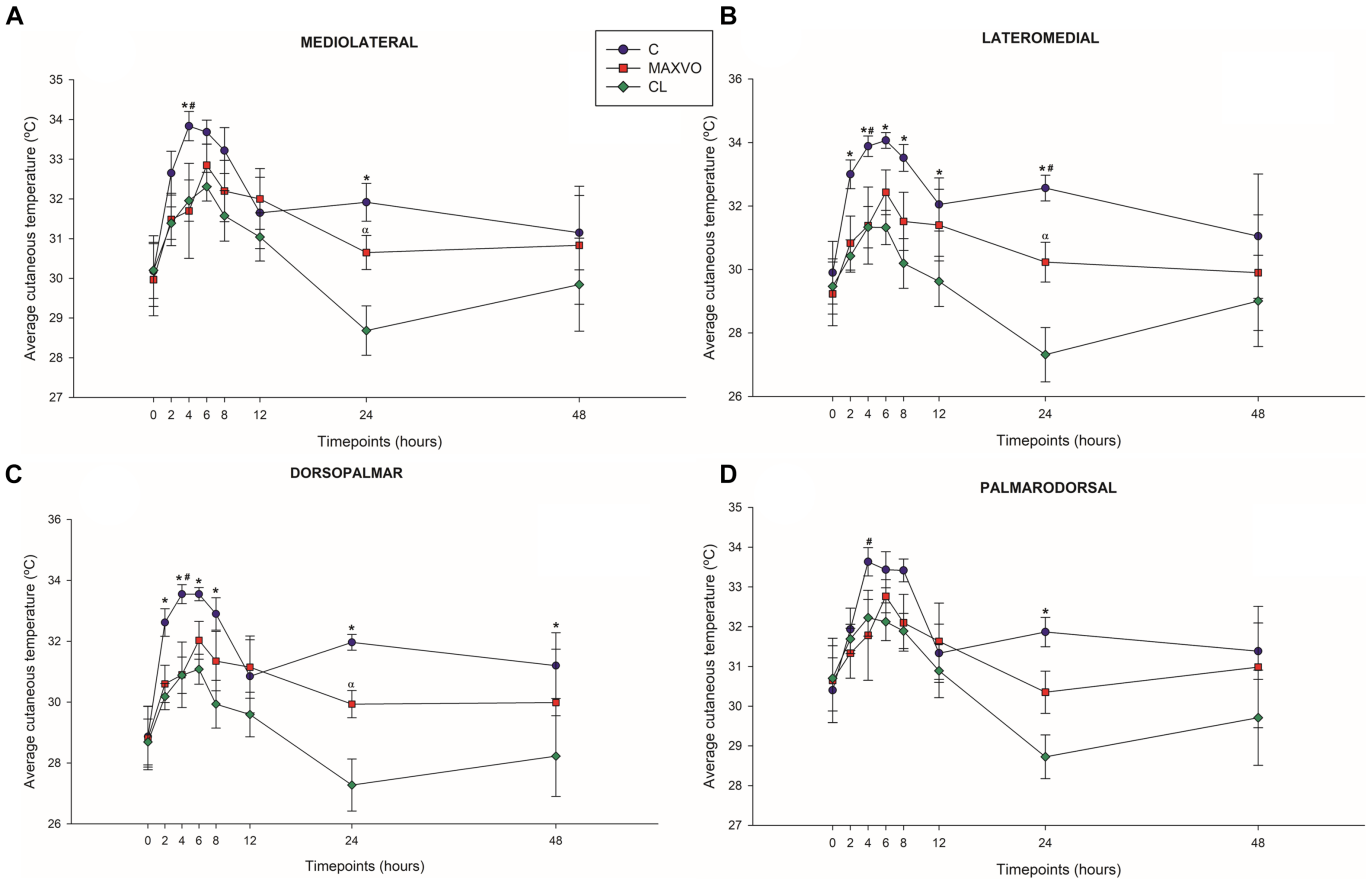
Graphical representation of means ± standard error of average cutaneous temperature of the **(A)** mediolateral, **(B)** lateromedial, **(C)** dorsopalmar and **(D)** palmarodorsal view of the carpal joint of horses submitted to experimental induction of transient synovitis for both trials (C and MAXVO). C, control group; MAXVO received meloxicam (Maxicam Gel^®^), administered orally at 0.6 mg/kg daily 3 days before LPS injection; CL, contralateral limbs (negative control). Synovitis induction by injection of the middle carpal joint with 10 EU LPS occurred at 0 h. ^#^Indicates difference between C and MAXVO. ^*^Indicates difference between C and CL. ^α^Indicates difference between CL and MAXVO. CL, contralateral limb (negative control). *p* < 0.05.

**Figure 4:**
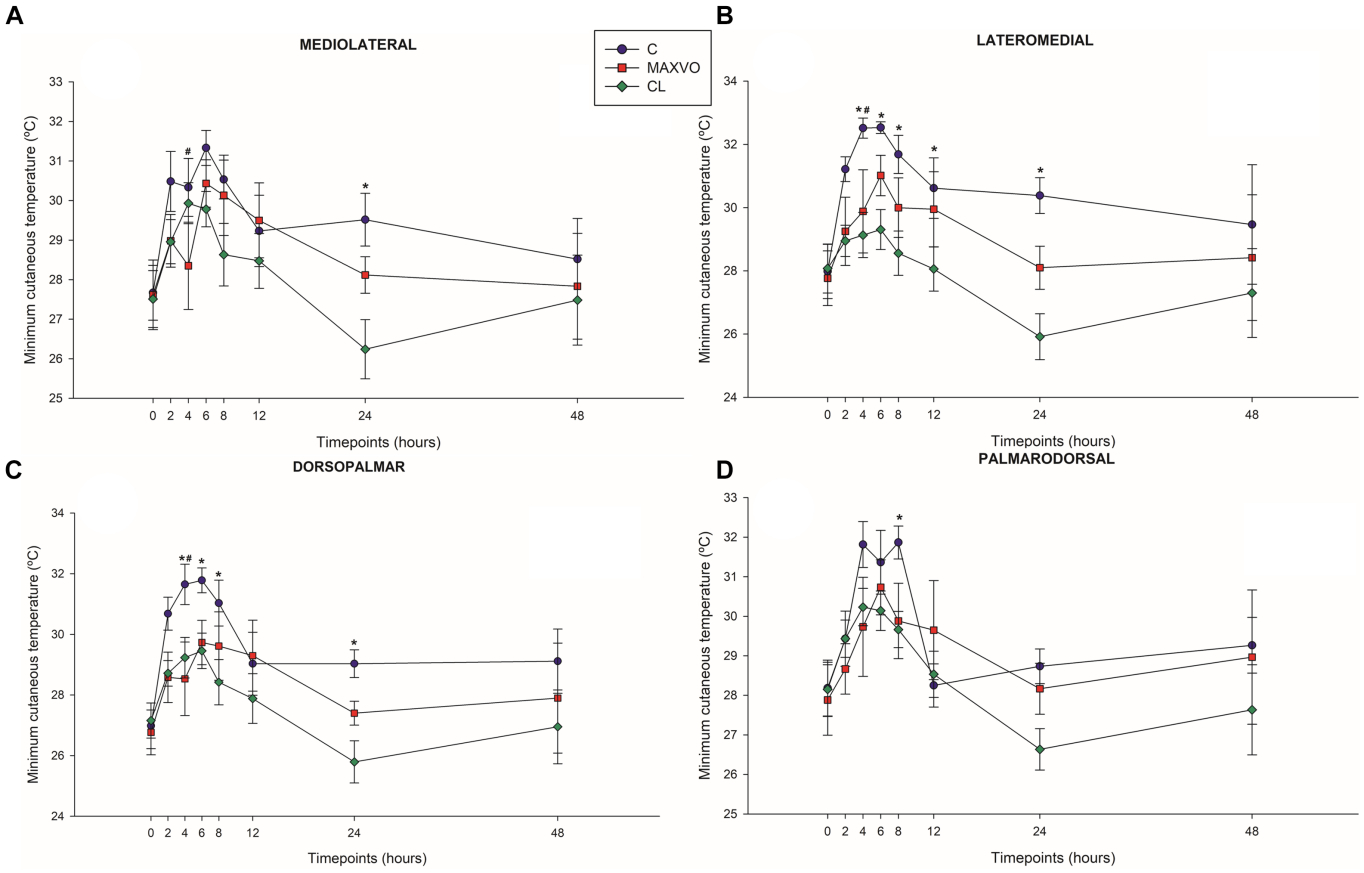
Graphical representation of means ± standard error of minimum cutaneous temperature of the **(A)** mediolateral, **(B)** lateromedial, **(C)** dorsopalmar and **(D)** palmarodorsal view of the carpal joint of horses submitted to experimental induction of transient synovitis for both trials (C and MAXVO). C, control group; MAXVO received meloxicam (Maxicam Gel^®^), administered orally at 0.6 mg/kg daily 3 days before LPS injection; CL, contralateral limbs (negative control). Synovitis induction by injection of the middle carpal joint with 10 EU LPS occurred at 0 h. ^#^Indicates difference between C and MAXVO. ^*^Indicates difference between C and CL. ^α^Indicates difference between CL and MAXVO. CL, contralateral limb (negative control). *p* < 0.05.

**Figure 5:**
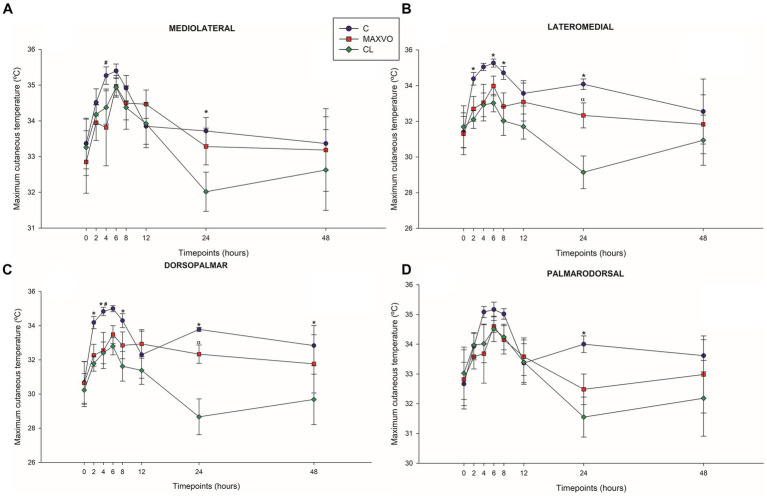
Graphical representation of means ± standard error of maximum cutaneous temperature of the **(A)** mediolateral, **(B)** lateromedial, **(C)** dorsopalmar and **(D)** palmarodorsal view of the carpal joint of horses submitted to experimental induction of transient synovitis for both trials (C and MAXVO). C, control group; MAXVO received meloxicam (Maxicam Gel^®^), administered orally at 0.6 mg/kg daily 3 days before LPS injection; CL, contralateral limbs (negative control). Synovitis induction by injection of the middle carpal joint with 10 EU LPS occurred at 0 h. ^#^Indicates difference between C and MAXVO. ^*^Indicates difference between C and CL. ^α^Indicates difference between CL and MAXVO. CL, contralateral limb (negative control). *p* < 0.05.

IRT was used to indirectly detect inflammation by measuring heat radiation. In this study, it was found that horses who received meloxicam had lower average, maximum, and minimum cutaneous temperatures 4 h post-injection in ML and DP views. Similarly, in the LM view, the MAXVO group showed lower average and minimum temperatures 4 h post-injection of LPS and lower average temperature 24 h post-injection compared to the control group. In the PD view, the MAXVO group showed lower average temperature 4 h post-injection of LPS. These findings suggest that meloxicam may have a positive impact on reducing cutaneous temperatures in horses post-LPS injection. It is worth noting that the cutaneous temperature in the group that received meloxicam was the same as the CL group most of the time, except at 24 h (average and maximum temperatures) after synovitis induction in ML, LM and DP views. In other words, the MAXVO group had the same cutaneous temperature as the contralateral limbs, which were not subjected to any invasive procedure (Figures 3–5 and Supplementary Tables S1–S3). In the study, it was found that the regions farthest from the LPS infiltration site, such as the ML and PD views, showed a higher temperature at the C group, when compared to the CL group only for the average temperature at 4 and 24 h. The PD view showed higher minimum and maximum temperatures only at 8 and 24 h, respectively, for the control group when compared to CL. At the ML view the C group showed higher maximum and minimum temperature than the CL only at 24 h. The Monte Carlo simulation was used to generate means and standard deviations, adjusted for ±2% accuracy, for each treatment and moment to ensure the reliability of our findings from the original data (Supplementary Tables S4–S15).

### 3.6 Multinomial logistic regression

**Figure 6:**
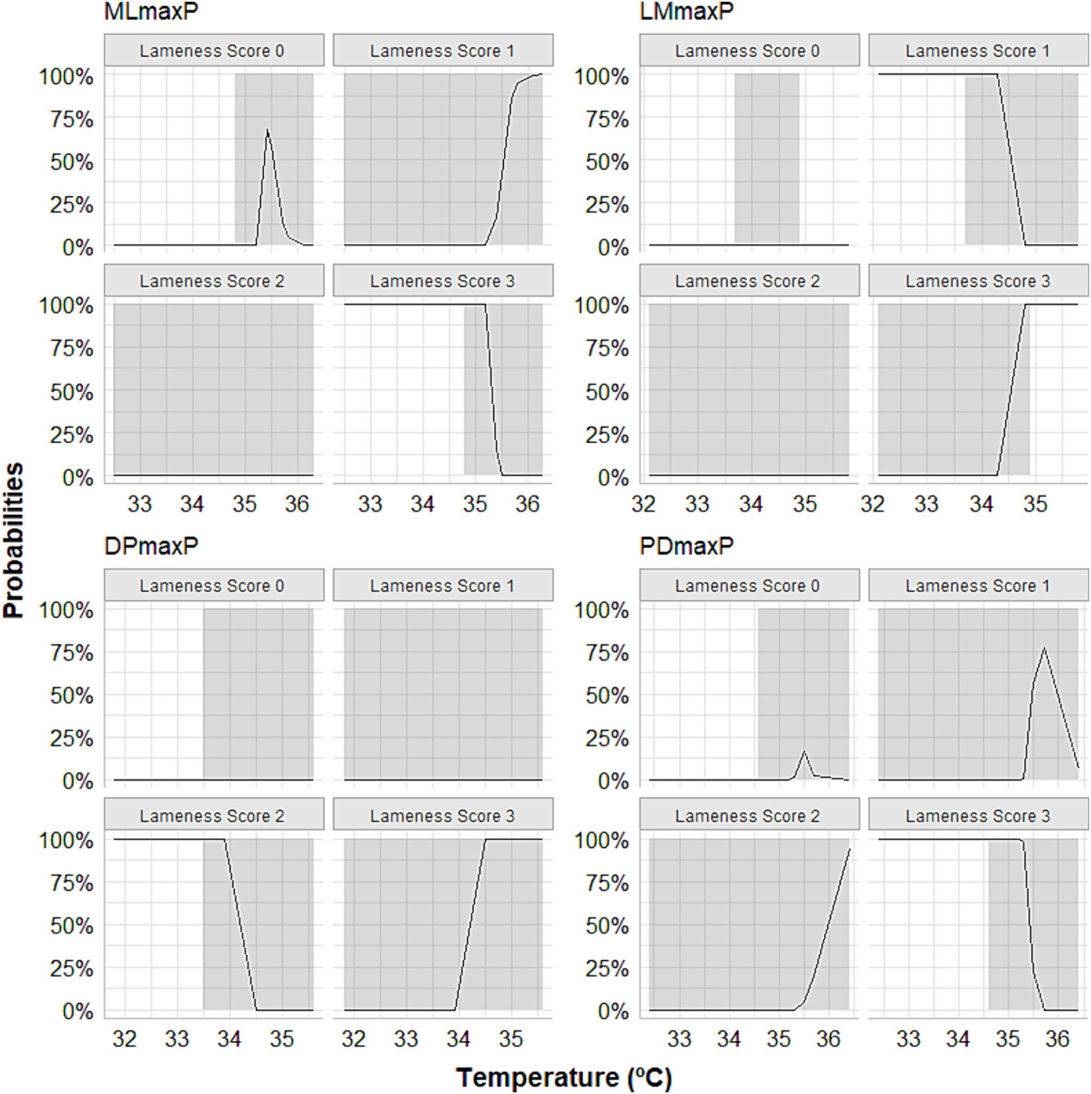
Curves depicting the calculation of predicted probabilities following multinomial logistic regression showing that the maximum temperature peaks observed in the lateromedial (LMmaxP) and dorsopalmar (DPmaxP) views could predict score 3 of lameness, especially when they exceeded 34°C. The horses were submitted to experimental induction of transient synovitis with 10 EU LPS and treated with meloxicam administered orally at 0.6 mg/kg daily 3 days before LPS injection.

Our MLR analysis revealed an association between the peaks of thermographic temperatures and lameness scores. The relative log odds, an equivalent to log odds in the logistic regression, demonstrated that a one-unit rise in the maximum temperature obtained by DP thermographic imaging is associated with an increase of 20 in the logarithm of the chances of having score 3 lameness compared to horses that presented lameness of score 0 (*p* < 0.0001). A significant association existed between a unit increase in maximum peak temperature obtained through LM thermographic imaging and a reduction of 18.8 in the logarithm of the odds of having a score 3 lameness compared to horses without lameness (*p* = 0.009). One unit increase in maximum peak temperature obtained by PD thermographic imaging is associated with an increase of 11.7 in the logarithm of the odds of having score 3 lameness compared to horses that do not lame (*p* < 0.0001). One unit increase in maximum temperature obtained by PD thermographic imaging is associated with a reduction of 18.7 in the logarithm of the odds of having score 3 lameness compared to horses that do not lame (*p* < 0.0001). The group of horses that received meloxicam orally is associated with a reduction of 76.2 in the logarithm of the odds of having grade 3 lameness compared to animals that do not lame (*p* < 0.0001). The relative risk ratios, equivalent to odds ratios in logistic regression, showed that one unit increase in the maximum temperature obtained by the thermographic image obtained from the DP view multiplies the chances of lameness classification 3 concerning horses that do not lame (score 0) by 4,528 (353%). Regarding the LM view, one unit increase in maximum temperature, obtained by thermal imaging, induced the chances of lameness, score 3, concerning horses that do not lame in 47.8%. The horses that received meloxicam, when compared with the control group, multiplied the chances of having a score of 1 lameness compared to animals that do not have lameness (score 0) by 146% (*p* < 0.0001). Lastly, the predicted probabilities, the same approach as logistic regression, but it is the probability of falling in a specific category, showed that the DP and LM positions’ maximum temperature peaks were able to predict degree 3 of lameness, especially above 34°C (Figure 6).

## 4 Discussion

Infrared thermography can be essential in diagnosing and treating inflammatory processes (15). This diagnostic technique indirectly determines the presence of a nociceptive stimulus through the activation of the nervous system and its vasomotor responses, leading to an inflammatory process and increased heat radiation (1). Thermography can be an important tool to measure the reduction of inflammation or to check the effectiveness of anti-inflammatory medication (14). Changes in local temperature of the middle carpal joint, in addition to the results of synovial fluid variables, such as TNCC, TP, and PGE_2_, confirmed the induction of synovitis. The present study showed that injecting 10 EU of *E. coli* O55:B5 LPS into the middle carpal joint of horses consistently induced transient synovitis and lameness. These findings are compatible with the results obtained in a previous study (11), which showed that a 10 EU dose of LPS induced lameness of clinically acceptable intensity, eliminating the need for additional doses of LPS.

It is important to highlight the use of the EU instead of mass units when processing and preparing LPS to ensure direct comparisons with other studies can be made (11). Standardizing laboratory procedures for preparing the LPS solution is also essential. Endotoxin molecules have micelle formation, which can affect their activity, so in this study, the stock and intermediate solutions were vortexed for 10 min at 1,500 rpm before dilution to break up LPS micelles and achieve a homogeneous solution. Additionally, the working solution was vortexed for 2 min at a speed of 1,500 rpm before induction, to disrupt any existing micelles (11).

This study evaluated the efficacy of oral preemptive meloxicam for inflammatory pain relief in horses with transient lameness and synovitis induced by the LPS model. The study findings suggest that this medication is a possible option for managing inflammation in horses in a preventative manner. Based on former studies, meloxicam treatment was initiated 3 days before LPS injection (10, 11). The researchers guided that the model is advantageous in providing information regarding the preemptive effects of treatments attributable to the concise duration of inflammation induced by LPS injection. This model provides relative data on whether it treats the signs of inflammation. Indeed, some of it is prevented when used in this manner. Therefore, the horses were given treatment for a few days before inducing synovitis. This decision was based on information regarding meloxicam pharmacokinetics, which indicated a maximum concentration time (*T*_max_) of 2.62 ± 1.88 h (range: 1.5–8 h) and an elimination half-life of 10.2 ± 3.0 h (42). Besides, after injection, the LPS effect can vary widely, so the decision was made to consider the peak lameness reported in the literature (9–11, 43). This preventive treatment is likely to be clinically applicable for pain management in horses undergoing arthroscopic surgery (8) or surgical trauma, especially in the presence of pre-operative pain. It may prevent the initiation of a cascade of events that sensitize peripheral and central pain networks, leading to long-lasting maladaptive pain (44).

During the trial, clinical, cardiorespiratory, and hematological assessments were conducted to ensure the horses’ health and to check for any signs of systemic illness, such as fever, tachycardia, and tachypnea, which may have been caused by the intra-articular administration of LPS. Physical examination variables remained within the reference range for horses, indicating no exacerbated systemic inflammatory response due to LPS infiltration. As expected, there was an increment in RT for both groups, with the MAXVO group showing lower RT 4 h after synovitis induction. This finding suggests that meloxicam may have potential antipyretic action. Variations in RR values were probably related to the time of day of the collection. HR, RR, and RT increases could be related to the release of inflammatory mediators due to LPS injection or pain (39, 43, 45, 46). However, these parameters alone are non-specific for determining the presence and severity of pain and can be influenced by external factors (47, 48). Some studies that induced synovitis in horses with LPS injection found no changes in HR, RR, and RT (10–12, 49). These differences may be related to the dose of endotoxin used (11). Also, systolic arterial pressure could be added to check for signs of systemic LPS or meloxicam effect (39).

The results of hematological exams revealed that the group of horses who did not receive meloxicam displayed neutrophilia. This finding may indicate the presence of inflammatory conditions and pathophysiologic feedback in the body (50–52). Furthermore, a decrease in total hemoglobin and hematocrit was also observed. These hematological responses in humans with septic arthritis suggest that the hematopoietic system may undergo changes during an infectious process (53). Further studies are necessary to determine whether similar results occur in horses.

The assessment of joint circumference did not indicate a clinical detection of effusion. This may be due to the inconsistent doses of LPS found in previous studies (11). A wide range of dosages have been used to induce synovitis and lameness with LPS, ranging from 0.125 ng to 5,000 ng of the same serotype of *E. coli* O55:B5. Some authors have reported swelling after the induction of synovitis by intra-articular administration of LPS (45, 46, 49, 54). It should be noted that the absence of effusion does not necessarily mean the absence of inflammation. Additionally, it is important to highlight that the behavior was similar between the groups.

When diagnosing synovitis, TNCC, TP, and PGE_2_ concentrations are the most essential variables to assess. In both groups, the dose of LPS used increased these inflammatory biomarkers over time, and there was no difference between groups. This lack of difference between groups may be attributed to the lower frequency of meloxicam administration in the current therapeutic protocol compared to former studies (10, 11). Another factor that could contribute to the increase in these synovial inflammatory markers is the impact of successive arthrocentesis over time, despite the mild impact of this repetitive procedure on synovial fluid cytology, even without clinical significance (11, 55). The intra-articular injection of LPS had a more significant impact on the synovial fluid composition than successive arthrocentesis (11, 43, 56). However, it has been suggested by former studies that the repeated aspiration of synovial fluid could potentially serve as a confounding factor when using synovial inflammatory biomarkers to diagnose joint diseases (57, 58).

The study followed established criteria (39) for determining lameness, using blinded subjective evaluation. The study found that the peak of lameness occurred 4 h after induction in the control group. However, for the horses that received meloxicam, there was no significant change in the degree of lameness after the injection of LPS. This result is consistent with another study that evaluated the experimental induction of transient lameness and synovitis in trotter breed horses treated with oral administration of meloxicam (11). This suggests that the investigational veterinary product tested in this study effectively reduces inflammation and pain. Additionally, quantitative gait analysis could complement visual lameness assessment, as it provides objective information to support clinical decision-making during lameness evaluations (59, 60).

Detecting and quantifying inflammation and joint pain in horses has been a topic of research for many years. The scores of lameness and severity of synovitis were reflected in the skin surface temperature of the middle carpal joint obtained via thermography. Temperature is a key physical property that can be quantified using infrared thermography. It directly provides information about the inflammatory component of joint diseases (15). The present study focuses on using blinded infrared thermographic examination to detect changes in skin temperature surface pattern as an indicator of the inflammatory response induced by transient synovitis. The study found that joint temperatures increased significantly in horses with synovitis, while the horses that did not receive meloxicam showed a marked increase in temperature. The skin surface temperature can be used as an accurate indicator of the physiological state, with changes in temperature being caused by changes in local perfusion. Thermographic representation is based on tissue vascularization and blood supply and inflammation causes an increase in blood supply, which increases the local temperature (13, 30).

The MAXVO group showed lower surface temperatures in comparison to the control group, which indicates less intense inflammation. Increased heat is one of the classic signs of the inflammatory process. This is because tissue injuries can cause vasodilation and an increase in exothermic cellular metabolism, leading to an increase in temperature at the site of the injury (61). Hence, it is possible to obtain quantitative data on the degree of joint inflammation by thermographically evaluating the cutaneous surface of the joints. This is done by measuring the infrared radiation emitted spontaneously by the site of the joint injury. Several studies have used this technique to evaluate joint inflammation (1, 15, 30, 31, 33, 38).

To accurately compare the left and right sides, capturing images of contralateral joints at an equal imaging distance in separate images is crucial (14). The contralateral joint was used as a negative control for both C and MAXVO. After inducing synovitis, the group that received meloxicam had a temperature of the middle carpal joint that was like that of the contralateral joint (negative control), which did not receive LPS and was always clinically normal. Only 24 h after the induction of synovitis MAXVO group presented higher cutaneous temperature than the negative control in all regions of interest, except in the PD view. This result indicates the effectiveness of meloxicam as an anti-inflammatory, as the skin temperature of the middle carpal joint that received the induction of inflammation in the group medicated with meloxicam remained like the joint that was not infiltrated with LPS at most timepoints.

MLR, a precise and reliable extension of binary logistic regression, applies to a categorical outcome variable with more than two categories. It was found that the predicted probabilities, like the approach used in logistic regression, can be used to determine the likelihood of falling into a specific category. In this case, this study found that the maximum temperature peaks of the DP and LM positions can be used to predict the severity of lameness with a high level of accuracy, especially when the temperature exceeds 34°C.

The results of this study have wide-ranging implications for the field of functional infrared thermography imaging and its use in lameness management in horses. Thermography could be a justifiable option to aid in the diagnosis of clinical or experimental synovitis, as its use could avoid sequential invasive procedures, such as sedation and intra-articular collection. Additionally, using thermography can prevent the promotion of an additional inflammatory process promoted by repeated aspiration of synovial fluid that could mask the actual effects of LPS or the action of NSAIDs. In this regard, IRT examination combined with lameness checking proved to be a more sensitive diagnostic tool to detect the effectiveness of the NSAID used herein. These findings could pave the way for future research and practical applications in this area, offering potential benefits for both human and animal health.

## 5 Conclusion

Horses that were given meloxicam orally before the induction of synovitis with LPS showed a notable decrease in the temperature of the middle carpal joint surface detected by infrared thermography. This reduction in temperature was accompanied by a significant mitigation of lameness. In conclusion, the use of Maxicam Gel^®^ proved to be an effective preventive anti-inflammatory medication for horses.

## Data availability statement

The original contributions presented in the study are included in the article/Supplementary material, further inquiries can be directed to the corresponding author.

## Ethics statement

The study adhered to the Ethical Principles in Animal Experimentation as established by the National Council for Control in Animal Experimentation (CONCEA). The protocol underwent review and approval by the Ethics Committee on the Use of Animals—CEUA—UNESP, Jaboticabal, Brazil (Protocol No. 2887/2021). The study was conducted in accordance with the local legislation and institutional requirements.

## Author contributions

JC: Software, Writing – review & editing, Writing – original draft, Investigation, Formal analysis, Data curation, Conceptualization. DD: Writing – review & editing, Writing – original draft, Methodology, Conceptualization, Supervision. TL: Writing – original draft, Software, Investigation, Formal analysis, Data curation. NS: Writing – original draft, Software, Investigation, Formal analysis, Data curation. AS: Writing – original draft, Software, Investigation, Formal analysis, Data curation. GRi: Writing – original draft, Investigation, Formal analysis. FA: Writing – review & editing, Conceptualization. BA: Writing – review & editing, Conceptualization. IG: Writing – original draft, Conceptualization. GRa: Writing – review & editing, Methodology, Formal analysis. GF: Writing – review & editing, Writing – original draft, Supervision, Resources, Project administration, Methodology, Investigation, Funding acquisition, Formal analysis, Conceptualization.
