# Supplementary material for: Functional infrared thermography imaging can be used to assess the effectiveness of Maxicam Gel® in pre-emptively treating transient synovitis and lameness in horses
Source: Front Vet Sci. 2024 Jun 11;11:1399815. doi: 10.3389/fvets.2024.1399815 (PMC11197459; doi:10.3389/fvets.2024.1399815)
Supplement: Supplementary file 1 [file Table_1.DOCX]

Supplementary Material

## Supplementary Figures


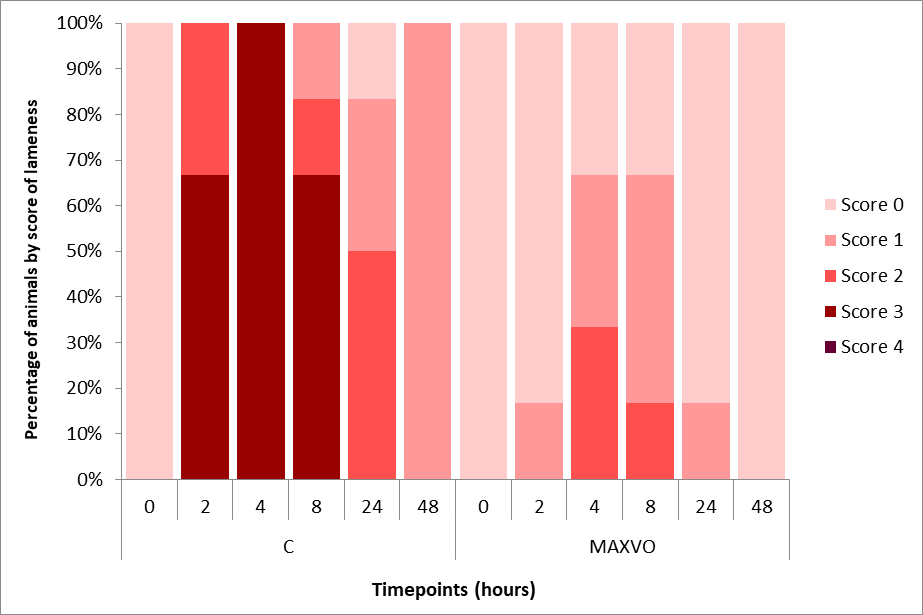


**Supplementary Figure 1.** Graphical representation of the degree of clinical lameness of horses submitted to experimental induction of transient synovitis for both trials (C and MAXVO). C=control group; MAXVO received meloxicam (Maxicam Gel®), administered orally at 0.6 mg/kg daily 3 days before LPS injection. Synovitis induction by injection of the middle carpal joint with 10 EU LPS occurred at 0h.

1. **Supplementary tables**

**Supplementary Table 1.** Means ± standard deviation of average cutaneous temperature of the mediolateral (ML), lateromedial (LM), dorsopalmar (DP) and palmarodorsal (PD) views of the carpal joint of horses submitted to experimental induction of transient synovitis for both trials (C and MAXVO). C=control group; MAXVO received meloxicam (Maxicam Gel®), administered orally at 0.6 mg/kg daily 3 days before LPS injection; CL=contralateral limbs (negative control). Synovitis induction by injection of the middle carpal joint with 10 EU LPS occurred at 0h.

| **Variable** | **Groups** | **Assessment times (hours)** | | | | | | | |
| --- | --- | --- | --- | --- | --- | --- | --- | --- | --- |
|  |  | **0** | **2** | **4** | **6** | **8** | **12** | **24** | **48** |
| **ML** | **C** | 30.2±2.2^a^ | 32.7±1.4^ab^ | 33.8±0.9^b^ | 33.7±0.7^b^ | 33.2±1.4^b^ | 31.7±2.2^ab^ | 31.9±1.2^ab^ | 31.2±2.3^a^ |
|  | **MAXVO** | 30.0±2.2^a^ | 31.5±1.6^ab^ | 31.7±2.9^ab^ | 32.9±1.3^b^ | 32.2±1.9^ab^ | 32.0±1.9^ab^ | 30.7±1.1^ab^ | 30.8±3.6^ab^ |
|  | **CL** | 30.2±1.7^ab^ | 31.4±1.0^a^ | 32.0±1.3^a^ | 32.3±0.9^a^ | 31.6±1.6^a^ | 31.0±1.5^ab^ | 28.7±1.5^b^ | 29.8±2.9^ab^ |
| **LM** | **C** | 29.9±2.4^a^ | 33.0±1.1^ab^ | 33.9±0.8^b^ | 34.1±0.6^b^ | 33.5±1.0^b^ | 32.1±2.0^ab^ | 32.6±1.0^ab^ | 31.0±4.8^ab^ |
|  | **MAXVO** | 29.2±2.5^a^ | 30.9±2.1^ab^ | 31.4±3.0^ab^ | 32.4±1.7^b^ | 31.5±2.3^ab^ | 31.4±2.8^ab^ | 30.2±1.5^ab^ | 29.9±4.5^ab^ |
|  | **CL** | 29.5±2.1^ab^ | 30.4±1.3^ab^ | 31.3±1.6^a^ | 31.3±1.3^a^ | 30.2±1.9^ab^ | 29.6±1.9^ab^ | 27.3±2.1^b^ | 29.0±3.5^ab^ |
| **DP** | **C** | 28.9±2.5^a^ | 32.6±1.1^b^ | 33.6±0.8^b^ | 33.6±0.5^b^ | 32.9±1.3^b^ | 30.9±2.9^ab^ | 32.0±0.6^b^ | 31.2±2.7^ab^ |
|  | **MAXVO** | 28.8±2.6^a^ | 30.6±1.5^ab^ | 30.9±2.6^ab^ | 32.0±1.5^b^ | 31.4±2.4^ab^ | 31.2±2.5^ab^ | 30.0±1.1^ab^ | 30.0±4.3^ab^ |
|  | **CL** | 28.7±1.9^ab^ | 30.2±1.1^ab^ | 30.9±1.5^a^ | 31.1±1.2^a^ | 29.9±1.9^ab^ | 29.6±1.8^ab^ | 27.3±2.1^b^ | 28.2±3.3^ab^ |
| **PD** | **C** | 30.4±2.0^a^ | 31.9±1.3^ab^ | 33.6±0.9^b^ | 33.4±1.1^b^ | 33.4±0.7^b^ | 31.3±1.8^ab^ | 31.9±0.9^ab^ | 31.4±1.7^ab^ |
|  | **MAXVO** | 30.7±2.6 | 31.3±1.5 | 31.8±2.8 | 32.8±1.0 | 32.1±1.8 | 31.6±2.4 | 30.4±1.3 | 31.0±3.7 |
|  | **CL** | 30.7±2.0^ab^ | 31.7±0.9^a^ | 32.2±1.1^a^ | 32.1±1.2^a^ | 31.9±1.1^a^ | 30.9±1.7^ab^ | 28.7±1.4^b^ | 29.7±2.9^ab^ |

**Supplementary Table 2.** Means ± standard deviation of maximum cutaneous temperature of the mediolateral (ML), lateromedial (LM), dorsopalmar (DP) and palmarodorsal (PD) views of the carpal joint of horses submitted to experimental induction of transient synovitis for both trials (C and MAXVO). C=control group; MAXVO received meloxicam (Maxicam Gel®), administered orally at 0.6 mg/kg daily 3 days before LPS injection; CL=contralateral limbs (negative control). Synovitis induction by injection of the middle carpal joint with 10 EU LPS occurred at 0h.

| **Variable** | **Groups** | **Assessment times (hours)** | | | | | | | |
| --- | --- | --- | --- | --- | --- | --- | --- | --- | --- |
|  |  | **0** | **2** | **4** | **6** | **8** | **12** | **24** | **48** |
| **ML** | **C** | 33.4±1.7 | 34.5±0.9 | 35.3±0.6 | 35.4±0.5 | 34.9±0.9 | 33.9±1.5 | 33.7±0.9 | 33.4±1.8 |
|  | **MAXVO** | 32.9±2.2^a^ | 34.0±1.2^ab^ | 33.8±2.6^ab^ | 35.0±0.7^b^ | 34.5±1.2^ab^ | 34.5±1.0^ab^ | 33.3±1.3^ab^ | 33.2±2.8^ab^ |
|  | **CL** | 33.3±1.9^abc^ | 34.2±0.7^ac^ | 34.4±1.1^ac^ | 34.9±0.6^c^ | 34.4±1.5^ac^ | 33.9±1.4^abc^ | 32.0±1.3^b^ | 32.6±2.8^ab^ |
| **LM** | **C** | 31.4±2.1^a^ | 34.4±0.9^ab^ | 35.1±0.5^b^ | 35.3±0.6^b^ | 34.7±0.9^b^ | 33.6±1.7^ab^ | 34.1±0.7^ab^ | 32.6±4.5^ab^ |
|  | **MAXVO** | 31.3±2.9 | 32.7±1.7 | 33.1±2.5 | 34.0±1.4 | 32.8±1.9 | 33.1±2.6 | 32.3±1.7 | 31.8±4.0 |
|  | **CL** | 31.7±2.9^ab^ | 32.1±1.2^ab^ | 32.9±1.6^a^ | 33.0±1.2^a^ | 32.0±2.0^ab^ | 31.7±1.7^ab^ | 29.1±2.2^b^ | 30.9±3.5^ab^ |
| **DP** | **C** | 30.7±3.0^a^ | 34.2±0.9^b^ | 34.8±0.6^b^ | 35.0±0.4^b^ | 34.3±1.0^b^ | 32.3±3.4^ab^ | 33.8±0.3^ab^ | 32.8±2.9^ab^ |
|  | **MAXVO** | 30.7±3.1 | 32.3±1.6 | 32.6±2.6 | 33.5±1.3 | 32.9±1.9 | 32.9±2.0 | 32.3±1.3 | 31.8±4.2 |
|  | **CL** | 30.2±2.4^ab^ | 31.8±1.1^ab^ | 32.4±1.6^a^ | 32.8±1.3^a^ | 31.6±2.1^ab^ | 31.4±2.0^ab^ | 28.7±2.6^b^ | 29.7±3.6^ab^ |
| **PD** | **C** | 32.7±1.8^a^ | 33.9±1.1^ab^ | 35.1±0.5^b^ | 35.2±0.6^b^ | 35.0±0.4^b^ | 33.4±1.6^ab^ | 34.0±0.7^ab^ | 33.6±1.3^ab^ |
|  | **MAXVO** | 32.8±2.4 | 33.6±1.0 | 33.7±2.4 | 34.6±0.5 | 34.2±1.2 | 33.6±1.6 | 32.5±1.3 | 33.0±3.2 |
|  | **CL** | 33.0±2.2^abc^ | 34.0±1.0^ac^ | 34.0±1.6^ac^ | 34.5±1.0^c^ | 34.2±1.1^ac^ | 33.4±1.8^abc^ | 31.6±1.7^b^ | 32.2±3.1^ab^ |

**Supplementary Table 3.** Means ± standard deviation of minimum cutaneous temperature of the mediolateral (ML), lateromedial (LM), dorsopalmar (DP) and palmarodorsal (PD) views of the carpal joint of horses submitted to experimental induction of transient synovitis for both trials (C and MAXVO). C=control group; MAXVO received meloxicam (Maxicam Gel®), administered orally at 0.6 mg/kg daily 3 days before LPS injection; CL=contralateral limbs (negative control). Synovitis induction by injection of the middle carpal joint with 10 EU LPS occurred at 0h.

| **Variable** | **Groups** | **Assessment times (hours)** | | | | | | | |
| --- | --- | --- | --- | --- | --- | --- | --- | --- | --- |
|  |  | **0** | **2** | **4** | **6** | **8** | **12** | **24** | **48** |
| **ML** | **C** | 27.7±1.7^a^ | 30.5±1.9^ab^ | 30.3±1.8^ab^ | 31.3±1.1^b^ | 30.5±1.2^b^ | 29.2±2.2^ab^ | 29.5±1.6^ab^ | 28.5±2.5^ab^ |
|  | **MAXVO** | 27.6±2.2 | 29.0±1.6 | 28.4±2.7 | 30.4±1.5 | 30.1±2.5 | 29.5±2.3 | 28.1±1.1 | 27.8±3.3 |
|  | **CL** | 27.5±1.8^ab^ | 29.0±1.4^ab^ | 29.9±1.3^a^ | 29.8±1.1^a^ | 28.6±1.9^ab^ | 28.5±1.7^ab^ | 26.2±1.8^b^ | 27.5±2.8^ab^ |
| **LM** | **C** | 28.0±2.1^a^ | 31.2±1.0^b^ | 32.5±0.8^b^ | 32.5±0.5^b^ | 31.7±1.5^b^ | 30.6±2.3^ab^ | 30.4±1.4^ab^ | 29.5±4.6^ab^ |
|  | **MAXVO** | 27.8±2.1^a^ | 29.3±2.7^ab^ | 29.9±3.2^ab^ | 31.0±1.6^b^ | 30.0±2.3^ab^ | 30.0±2.9^ab^ | 28.1±1.7^ab^ | 28.4±4.9^ab^ |
|  | **CL** | 28.1±1.9^ab^ | 29.0±1.2^ab^ | 29.1±1.8^ab^ | 29.3±1.6^a^ | 28.6±1.7^ab^ | 28.1±1.7^ab^ | 25.9±1.8^b^ | 27.3±3.5^ab^ |
| **DP** | **C** | 27.0±1.9^a^ | 30.7±1.3^b^ | 31.7±1.6^b^ | 31.8±1.0^b^ | 31.0±1.9^b^ | 29.0±2.6^ab^ | 29.0±1.1^ab^ | 29.1±2.6^ab^ |
|  | **MAXVO** | 26.8±1.8 | 28.6±2.0 | 28.5±3.0 | 29.7±1.8 | 29.6±2.8 | 29.3±2.9 | 27.4±1.0 | 27.9±4.4 |
|  | **CL** | 27.2±1.4^ab^ | 28.7±1.0^ab^ | 29.2±1.6^a^ | 29.5±1.4^a^ | 28.4±1.8^ab^ | 27.9±2.0^ab^ | 25.8±1.7^b^ | 27.0±3.0^ab^ |
| **PD** | **C** | 28.2±1.7^ac^ | 29.4±1.7^abc^ | 31.8±1.4^b^ | 31.4±2.0^bd^ | 31.9±1.0^b^ | 28.3±1.3^c^ | 28.7±1.1^cd^ | 29.3±1.7^abc^ |
|  | **MAXVO** | 27.9±2.2 | 28.7±1.6 | 29.7±3.1 | 30.7±1.7 | 29.9±2.3 | 29.7±3.1 | 28.2±1.6 | 29.0±4.2 |
|  | **CL** | 28.2±1.7^ab^ | 29.4±1.2^ab^ | 30.2±1.2^a^ | 30.1±1.2^a^ | 29.7±1.1^a^ | 28.5±1.4^ab^ | 26.7±1.3^b^ | 27.6±2.8^ab^ |

**Supplementary Table 4.** Simulated and adjusted means ± standard deviation of average cutaneous temperature of the mediolateral (ML) view from the Monte Carlo simulation. The horses were submitted to experimental induction of transient synovitis for both trials (C and MAXVO). C=control group; MAXVO received meloxicam (Maxicam Gel®), administered orally at 0.6 mg/kg daily three days before LPS injection; CL=contralateral limbs (negative control). The means and standard deviation of each treatment and moment were taken from the original data. The simulation was also adjusted for ± 2% accuracy.

| **Groups (*n*=100)** | **Timepoints (hours)** | | | | | | | |
| --- | --- | --- | --- | --- | --- | --- | --- | --- |
|  | **0** | **2** | **4** | **6** | **8** | **12** | **24** | **48** |
| **C_SIM_** | 30.0±1.33 | 32.7±1.14 | 33.8±0.98 | 33.7±0.85 | 33.1±1.08 | 31.8±1.61 | 31.8±1.02 | 31.2±1.61 |
| **C_ADJ2%_** | 30.2±1.00 | 32.7±0.82 | 33.8±0.82 | 33.6±0.59 | 33.2±0.81 | 31.6±1.09 | 32.0±0.84 | 31.2±1.08 |
| ***p-value*** | 0.3578 | 0.5896 | 0.9607 | 0.3195 | 0.4997 | 0.3284 | 0.2316 | 0.9067 |
| **MAXVO_SIM_** | 30.0±1.53 | 31.9±1.17 | 31.8±1.64 | 32.8±1.17 | 32.3±1.32 | 31.9±1.37 | 30.7±1.01 | 30.9±1.77 |
| **MAXVO_ADJ2%_** | 30.2±1.08 | 31.6±0.92 | 31.8±1.04 | 32.8±0.78 | 32.2±0.95 | 32.0±1.01 | 30.6±0.78 | 30.8± 1.27 |
| ***p-value*** | 0.4073 | 0.1801 | 0.9051 | 0.8357 | 0.6839 | 0.4081 | 0.3763 | 0.7593 |
| **CL_SIM_** | 30.3±1.35 | 31.5±0.96 | 31.2±1.15 | 32.1±1.02 | 31.6±1.18 | 31.2±1.21 | 28.7±1.21 | 29.9±1.74 |
| **CL_ADJ2%_** | 30.2±0.99 | 31.4±0.77 | 31.9±0.80 | 32.3±0.62 | 31.4±0.92 | 31.0±0.80 | 28.5±0.86 | 29.9±1.24 |
| ***p-value*** | 0.5815 | 0.5167 | 0.4522 | 0.0460 | 0.3658 | 0.1750 | 0.2177 | 0.8251 |

**Supplementary Table 5.** Simulated and adjusted means ± standard deviation of average cutaneous temperature of the lateromedial (LM) view from the Monte Carlo simulation. The horses were submitted to experimental induction of transient synovitis for both trials (C and MAXVO). C=control group; MAXVO received meloxicam (Maxicam Gel®), administered orally at 0.6 mg/kg daily three days before LPS injection; CL=contralateral limbs (negative control). The means and standard deviation of each treatment and moment were taken from the original data. The simulation was also adjusted for ± 2% accuracy.

| **Groups (*n*=100)** | **Timepoints (hours)** | | | | | | | |
| --- | --- | --- | --- | --- | --- | --- | --- | --- |
|  | **0** | **2** | **4** | **6** | **8** | **12** | **24** | **48** |
| **C_SIM_** | 29.7±1.57 | 33.3±1.64 | 34.0±0.89 | 34.0±0.68 | 33.5±0.99 | 31.2±1.38 | 32.5±1.02 | 31.2±2.17 |
| **C_ADJ2%_** | 28.8±1.13 | 32.9±1.17 | 33.9±0.71 | 34.0±0.49 | 33.6±0.72 | 32.2±0.94 | 32.7±0.73 | 31.1±1.41 |
| ***p-value*** | 0.0001 | 0.0366 | 0.4848 | 0.6015 | 0.7528 | 0.2772 | 0.3674 | 0.7675 |
| **MAXVO_SIM_** | 29.2±1.67 | 31.1±1.47 | 30.9±1.61 | 31.6±1.67 | 32.6±1.35 | 31.5±1.48 | 30.1±1.31 | 29.5±2.42 |
| **MAXVO_ADJ2%_** | 29.3±1.07 | 30.8±1.02 | 30.8±0.98 | 31.4±1.20 | 32.3±0.95 | 31.5±1.10 | 30.2±0.99 | 29.9±1.67 |
| ***p-value*** | 0.4624 | 0.4847 | 0.0825 | 0.0965 | 0.5869 | 0.2248 | 0.5166 | 0.2461 |
| **CL_SIM_** | 29.5±1.49 | 30.4±1.16 | 31.5±1.30 | 31.2±1.15 | 30.3±1.41 | 29.7±1.30 | 27.3±1.43 | 28.9±1.81 |
| **CL_ADJ2%_** | 29.4±1.18 | 30.5±0.74 | 31.2±0.89 | 31.5±0.83 | 30.2±0.96 | 29.5±1.08 | 27.1±0.99 | 29.1±1.36 |
| ***p-value*** | 0.4624 | 0.4624 | 0.4624 | 0.4624 | 0.4624 | 0.4624 | 0.4624 | 0.4624 |

**Supplementary Table 6.** Simulated and adjusted means ± standard deviation of average cutaneous temperature of the dorsopalmar (DP) view from the Monte Carlo simulation. The horses were submitted to experimental induction of transient synovitis for both trials (C and MAXVO). C=control group; MAXVO received meloxicam (Maxicam Gel®), administered orally at 0.6 mg/kg daily three days before LPS injection; CL=contralateral limbs (negative control). The means and standard deviation of each treatment and moment were taken from the original data. The simulation was also adjusted for ± 2% accuracy.

| **Groups (*n*=100)** | **Timepoints (hours)** | | | | | | | |
| --- | --- | --- | --- | --- | --- | --- | --- | --- |
|  | **0** | **2** | **4** | **6** | **8** | **12** | **24** | **48** |
| **C_SIM_** | 28.8 ±1.43 | 32.9±1.59 | 33.7±0.85 | 33.6±0.68 | 33.0±1.13 | 30.8±1.87 | 32.0±0.73 | 31.0±1.51 |
| **C_ADJ2%_** | 28.9±1.08 | 32.6±1.00 | 33.5±0.65 | 33.6±0.47 | 32.8±0.72 | 30.7±1.19 | 32.0±0.58 | 31.0±1.16 |
| ***p-value*** | 0.8172 | 0.1500 | 0.1598 | 0.8935 | 0.0753 | 0.7913 | 0.8222 | 0.7921 |
| **MAXVO_SIM_** | 29.0±1.56 | 30.6±1.23 | 30.8±1.76 | 32.1±1.18 | 31.3±1.56 | 31.1±1.50 | 29.9±1.1 | 29.9±2.51 |
| **MAXVO_ADJ2%_** | 29.0±1.16 | 30.7±0.89 | 30.8±1.22 | 32.0±0.92 | 31.3±1.18 | 31.1±1.20 | 30.0±0.78 | 30.0±1.53 |
| ***p-value*** | 0.9200 | 0.4766 | 0.9551 | 0.4450 | 0.8026 | 0.9053 | 0.6224 | 0.7524 |
| **CL_SIM_** | 28.6±1.30 | 30.1±0.98 | 30.9±1.22 | 31.1±1.05 | 30.0±1.25 | 29.6±1.42 | 27.2±1.57 | 28.0±1.87 |
| **CL_ADJ2%_** | 28.5±1.01 | 30.2±0.66 | 30.9±0.85 | 31.1±0.87 | 30.1±1.04 | 29.6±0.96 | 27.2±0.94 | 27.9±1.38 |
| ***p-value*** | 0.6852 | 0.4497 | 0.9058 | 0.9501 | 0.8009 | 0.7796 | 0.9209 | 0.7496 |

**Supplementary Table 7.** Simulated and adjusted means ± standard deviation of average cutaneous temperature of the palmarodorsal (PD) view from the Monte Carlo simulation. The horses were submitted to experimental induction of transient synovitis for both trials (C and MAXVO). C=control group; MAXVO received meloxicam (Maxicam Gel®), administered orally at 0.6 mg/kg daily three days before LPS injection; CL=contralateral limbs (negative control). The means and standard deviation of each treatment and moment were taken from the original data. The simulation was also adjusted for ± 2% accuracy.

| **Groups (*n*=100)** | **Timepoints (hours)** | | | | | | | |
| --- | --- | --- | --- | --- | --- | --- | --- | --- |
|  | **0** | **2** | **4** | **6** | **8** | **12** | **24** | **48** |
| **C_SIM_** | 30.4±1.39 | 31.9±1.01 | 33.5±0.91 | 33.5±1.10 | 33.5±0.75 | 31.1±1.23 | 31.9±0.79 | 31.6±1.09 |
| **C_ADJ2%_** | 30.6±0.89 | 31.9±0.76 | 33.5±0.60 | 33.4±0.75 | 33.3±0.55 | 31.3±0.97 | 31.8±0.66 | 31.4±0.80 |
| ***p-value*** | 0.1888 | 0.5195 | 0.9136 | 0.5898 | 0.0339 | 0.0956 | 0.3147 | 0.1697 |
| **MAXVO_SIM_** | 30.6±1.57 | 31.4±1.22 | 32.1±1.59 | 32.8±1.03 | 31.8±1.37 | 31.3±1.54 | 30.2±1.25 | 30.8±1.81 |
| **MAXVO_ADJ2%_** | 30.7±1.12 | 31.3±0.99 | 31.6±1.15 | 32.8±0.62 | 32.2±0.92 | 31.6±1.12 | 30.3±0.75 | 30.8±1.28 |
| ***p-value*** | 0.4029 | 0.3058 | 0.6457 | 0.497 | 0.0294 | 0.1623 | 0.4247 | 0.9279 |
| **CL_SIM_** | 30.9±1.47 | 31.7±0.92 | 32.2±1.11 | 32.0±1.10 | 32.0±1.04 | 30.7±1.16 | 28.5±1.03 | 29.6±1.72 |
| **CL_ADJ2%_** | 30.7±0.94 | 31.7±0.63 | 32.9±0.76 | 32.1±0.75 | 31.9±0.74 | 30.8±0.83 | 28.6±0.78 | 29.7±1.21 |
| ***p-value*** | 0.4614 | 0.7523 | 0.8024 | 0.1999 | 0.0795 | 0.4476 | 0.1865 | 0.5229 |

**Supplementary Table 8.** Simulated and adjusted means ± standard deviation of maximum cutaneous temperature of the mediolateral (ML) view from the Monte Carlo simulation. The horses were submitted to experimental induction of transient synovitis for both trials (C and MAXVO). C=control group; MAXVO received meloxicam (Maxicam Gel®), administered orally at 0.6 mg/kg daily three days before LPS injection; CL=contralateral limbs (negative control). The means and standard deviation of each treatment and moment were taken from the original data. The simulation was also adjusted for ± 2% accuracy.

| **Groups (*n*=100)** | **Timepoints (hours)** | | | | | | | |
| --- | --- | --- | --- | --- | --- | --- | --- | --- |
|  | **0** | **2** | **4** | **6** | **8** | **12** | **24** | **48** |
| **C_SIM_** | 33.4±1.22 | 34.5±0.89 | 35.3±0.79 | 35.4±0.71 | 34.9±1.00 | 33.9±1.15 | 33.7±0.91 | 33.2±1.38 |
| **C_ADJ2%_** | 33.4±0.94 | 34.4±0.66 | 35.2±0.56 | 35.4±0.52 | 35.0±0.64 | 34.0±0.90 | 33.7±0.67 | 33.4±0.93 |
| ***p-value*** | 0.9153 | 0.8632 | 0.5572 | 0.7901 | 0.5000 | 0.5892 | 0.7062 | 0.2479 |
| **MAXVO_SIM_** | 32.9±1.34 | 33.8±1.27 | 33.9±1.45 | 34.9±0.81 | 34.6±1.02 | 34.5±1.08 | 33.7±1.05 | 33.3±1.88 |
| **MAXVO_ADJ2%_** | 32.7±1.00 | 33.8±0.74 | 33.8±1.18 | 35.0±0.61 | 34.5±0.72 | 34.4±0.68 | 33.3±0.77 | 33.2±1.13 |
| ***p-value*** | 0.4723 | 0.8190 | 0.5391 | 0.5108 | 0.2137 | 0.3657 | 0.5059 | 0.5543 |
| **CL_SIM_** | 33.1±1.17 | 34.1±0.77 | 34.5±1.01 | 34.9±0.79 | 34.6±1.09 | 34.0±1.16 | 31.9±1.17 | 33.1±1.17 |
| **CL_ADJ2%_** | 33.3±0.91 | 34.2±0.59 | 34.3±0.81 | 34.9±0.57 | 34.5±0.84 | 34.0±0.85 | 32.0±0.78 | 33.3±0.91 |
| ***p-value*** | 0.1399 | 0.6940 | 0.1304 | 0.6593 | 0.262 | 0.5980 | 0.5638 | 0.1399 |

**Supplementary Table 9.** Simulated and adjusted means ± standard deviation of maximum cutaneous temperature of the lateromedial (LM) view from the Monte Carlo simulation. The horses were submitted to experimental induction of transient synovitis for both trials (C and MAXVO). C=control group; MAXVO received meloxicam (Maxicam Gel®), administered orally at 0.6 mg/kg daily three days before LPS injection; CL=contralateral limbs (negative control). The means and standard deviation of each treatment and moment were taken from the original data. The simulation was also adjusted for ± 2% accuracy.

| **Groups (*n*=100)** | **Timepoints (hours)** | | | | | | | |
| --- | --- | --- | --- | --- | --- | --- | --- | --- |
|  | **0** | **2** | **4** | **6** | **8** | **12** | **24** | **48** |
| **C_SIM_** | 31.4±1.44 | 34.3±0.95 | 35.0±0.63 | 35.2±0.77 | 34.5±0.93 | 33.8±1.35 | 34.0±0.91 | 32.6±2.04 |
| **C_ADJ2%_** | 31.3±1.00 | 34.4±0.62 | 35.1±0.51 | 35.3±0.57 | 34.6±0.69 | 33.6±0.92 | 34.0±0.64 | 32.5±1.80 |
| ***p-value*** | 0.5030 | 0.9596 | 0.8452 | 0.3700 | 0.3717 | 0.5030 | 0.9596 | 0.8452 |
| **MAXVO_SIM_** | 31.4±1.65 | 32.7±1.31 | 32.8±1.56 | 34.0±1.12 | 32.7±1.31 | 33.2±1.73 | 32.2±1.3 | 31.4±1.86 |
| **MAXVO_ADJ2%_** | 31.3±1.13 | 32.5±0.94 | 33.1±1.15 | 33.8±0.76 | 33.0±0.96 | 33.0±1.23 | 32.2±0.93 | 31.9±1.41 |
| ***p-value*** | 0.6021 | 0.2732 | 0.0724 | 0.1922 | 0.0298 | 0.2863 | 0.9568 | 0.0403 |
| **CL_SIM_** | 31.8±1.44 | 32.2±1.07 | 32.9±1.28 | 33.2±1.11 | 31.9±1.26 | 31.6±1.31 | 28.9±1.41 | 31.2±1.77 |
| **CL_ADJ2%_** | 31.5±1.29 | 32.1±0.82 | 32.9±0.89 | 33.1±0.73 | 32.0±0.98 | 31.8±1.04 | 29.1±1.13 | 30.8±1.18 |
| ***p-value*** | 0.1535 | 0.3065 | 0.8703 | 0.3999 | 0.6144 | 0.2971 | 0.1310 | 0.0533 |

**Supplementary Table 10.** Simulated and adjusted means ± standard deviation of maximum cutaneous temperature of the dorsopalmar (DP) view from the Monte Carlo simulation. The horses were submitted to experimental induction of transient synovitis for both trials (C and MAXVO). C=control group; MAXVO received meloxicam (Maxicam Gel®), administered orally at 0.6 mg/kg daily three days before LPS injection; CL=contralateral limbs (negative control). The means and standard deviation of each treatment and moment were taken from the original data. The simulation was also adjusted for ± 2% accuracy.

| **Groups (*n*=100)** | **Timepoints (hours)** | | | | | | | |
| --- | --- | --- | --- | --- | --- | --- | --- | --- |
|  | **0** | **2** | **4** | **6** | **8** | **12** | **24** | **48** |
| **C_SIM_** | 30.4±2.02 | 34.2±0.89 | 34.8±0.80 | 35.0±0.61 | 34.2±1.02 | 32.6±1.96 | 33.7±0.55 | 32.6±1.92 |
| **C_ADJ2%_** | 30.7±1.28 | 34.2±0.59 | 34.7±0.54 | 35.0±0.46 | 34.3±0.82 | 32.2±1.34 | 33.8±0.38 | 32.9±1.23 |
| ***p-value*** | 0.2783 | 0.3863 | 0.8928 | 0.6238 | 0.3883 | 0.1321 | 0.0610 | 0.2120 |
| **MAXVO_SIM_** | 31.1±1.72 | 32.3±1.23 | 32.6±1.60 | 33.4±1.03 | 32.8±1.34 | 33.0±1.45 | 32.2±0.98 | 31.2±1.94 |
| **MAXVO_ADJ2%_** | 30.7±1.38 | 32.1±0.76 | 32.6±1.11 | 33.5±0.74 | 32.9±1.01 | 32.9±1.00 | 32.2±0.93 | 31.7±1.28 |
| ***p-value*** | 0.0585 | 0.1613 | 0.9695 | 0.3249 | 0.4634 | 0.4920 | 0.8442 | 0.0378 |
| **CL_SIM_** | 30.2±1.39 | 31.8±1.21 | 32.5±1.12 | 32.7±1.13 | 31.5±1.49 | 31.2±1.47 | 28.6±1.48 | 29.5±1.86 |
| **CL_ADJ2%_** | 30.1±1.12 | 31.9±0.66 | 32.6±0.81 | 32.9±0.78 | 31.7±0.94 | 31.5±1.03 | 28.8±1.14 | 29.6±1.33 |
| ***p-value*** | 0.6665 | 0.3870 | 0.5420 | 0.1789 | 0.1075 | 0.2138 | 0.4751 | 0.7186 |

**Supplementary Table 11.** Simulated and adjusted means ± standard deviation of maximum cutaneous temperature of the palmarodorsal (PD) view from the Monte Carlo simulation. The horses were submitted to experimental induction of transient synovitis for both trials (C and MAXVO). C=control group; MAXVO received meloxicam (Maxicam Gel®), administered orally at 0.6 mg/kg daily three days before LPS injection; CL=contralateral limbs (negative control). The means and standard deviation of each treatment and moment were taken from the original data. The simulation was also adjusted for ± 2% accuracy.

| **Groups (*n*=100)** | **Timepoints (hours)** | | | | | | | |
| --- | --- | --- | --- | --- | --- | --- | --- | --- |
|  | **0** | **2** | **4** | **6** | **8** | **12** | **24** | **48** |
| **C_SIM_** | 32.6±1.38 | 33.8±1.08 | 35.0±0.73 | 35.0±0.82 | 35.0±0.64 | 33.4±1.23 | 34.0±0.80 | 33.5±1.22 |
| **C_ADJ2%_** | 32.8±0.97 | 34.0±0.64 | 35.1±0.52 | 35.2±0.57 | 35.0±0.51 | 33.3±0.80 | 34.1±0.48 | 33.6±0.84 |
| ***p-value*** | 0.2740 | 0.0932 | 0.2125 | 0.0503 | 0.8490 | 0.7225 | 0.6764 | 0.7893 |
| **MAXVO_SIM_** | 32.8±1.64 | 33.6±1.02 | 33.6±1.71 | 34.7±0.73 | 34.0±1.00 | 33.7±1.05 | 32.4±1.24 | 33.0±1.79 |
| **MAXVO_ADJ2%_** | 32.8±0.98 | 33.5±0.72 | 33.7±1.11 | 34.5±0.54 | 34.2±0.71 | 33.6±0.86 | 32.4±0.78 | 33.0±1.28 |
| ***p-value*** | 0.9161 | 0.4199 | 0.7165 | 0.0194 | 0.2914 | 0.4490 | 0.8175 | 0.9571 |
| **CL_SIM_** | 33.1±1.39 | 33.9±1.06 | 33.5±1.38 | 34.7±0.97 | 34.1±0.94 | 33.2±1.46 | 31.6±1.22 | 32.6±1.70 |
| **CL_ADJ2%_** | 33.0±1.00 | 34.0±0.72 | 34.1±1.01 | 34.6±0.72 | 34.2±0.67 | 33.4±0.96 | 31.6±0.77 | 32.3±1.18 |
| ***p-value*** | 0.5974 | 0.2082 | 0.3904 | 0.5749 | 0.4746 | 0.2302 | 0.6914 | 0.1018 |

**Supplementary Table 12.** Simulated and adjusted means ± standard deviation of minimum cutaneous temperature of the mediolateral (ML) view from the Monte Carlo simulation. The horses were submitted to experimental induction of transient synovitis for both trials (C and MAXVO). C=control group; MAXVO received meloxicam (Maxicam Gel®), administered orally at 0.6 mg/kg daily three days before LPS injection; CL=contralateral limbs (negative control). The means and standard deviation of each treatment and moment were taken from the original data. The simulation was also adjusted for ± 2% accuracy.

| **Groups (*n*=100)** | **Timepoints (hours)** | | | | | | | |
| --- | --- | --- | --- | --- | --- | --- | --- | --- |
|  | **0** | **2** | **4** | **6** | **8** | **12** | **24** | **48** |
| **C_SIM_** | 27.7±1.36 | 30.4±1.37 | 30.6±1.51 | 31.3±0.95 | 30.7±1.00 | 29.2±1.51 | 29.4±1.03 | 28.6±1.61 |
| **C_ADJ2%_** | 27.6±0.77 | 30.3±0.98 | 30.4±0.94 | 31.2±0.82 | 30.3±0.75 | 29.4±0.98 | 29.6±0.93 | 28.9±1.31 |
| ***p-value*** | 0.5418 | 0.4714 | 0.4009 | 0.4989 | 0.0003 | 0.3396 | 0.3431 | 0.6126 |
| **MAXVO_SIM_** | 27.7±1.43 | 29.1±1.20 | 29.1±1.20 | 30.3±1.14 | 30.3±1.50 | 29.5±1.55 | 27.8±1.10 | 27.7±1.79 |
| **MAXVO_ADJ2%_** | 27.7±0.81 | 28.9±1.01 | 28.9±1.01 | 30.5±0.85 | 30.0±0.99 | 29.5±1.00 | 28.1±0.69 | 27.8±1.37 |
| ***p-value*** | 0.7152 | 0.2909 | 0.2909 | 0.2206 | 0.1176 | 0.6542 | 0.0238 | 0.6718 |
| **CL_SIM_** | 27.6±1.48 | 28.9±1.16 | 29.7±1.09 | 29.8±0.98 | 28.5±1.36 | 28.5±1.20 | 26.3±1.22 | 27.5±1.71 |
| **CL_ADJ2%_** | 27.6±0.99 | 28.9±0.79 | 29.9±0.76 | 29.7±0.74 | 28.6±0.95 | 28.4±0.86 | 26.3±0.99 | 27.4±1.22 |
| ***p-value*** | 0.8600 | 0.9644 | 0.1762 | 0.9409 | 0.5505 | 0.5929 | 0.9217 | 0.6153 |

**Supplementary Table 13.** Simulated and adjusted means ± standard deviation of minimum cutaneous temperature of the lateromedial (LM) view from the Monte Carlo simulation. The horses were submitted to experimental induction of transient synovitis for both trials (C and MAXVO). C=control group; MAXVO received meloxicam (Maxicam Gel®), administered orally at 0.6 mg/kg daily three days before LPS injection; CL=contralateral limbs (negative control). The means and standard deviation of each treatment and moment were taken from the original data. The simulation was also adjusted for ± 2% accuracy.

| **Groups (*n*=100)** | **Timepoints (hours)** | | | | | | | |
| --- | --- | --- | --- | --- | --- | --- | --- | --- |
|  | **0** | **2** | **4** | **6** | **8** | **12** | **24** | **48** |
| **C_SIM_** | 28.1±1.28 | 31.1±1.03 | 32.5±0.83 | 32.5±0.63 | 31.7±1.31 | 30.7±1.58 | 30.3±1.14 | 29.3±2.08 |
| **C_ADJ2%_** | 28.0±0.96 | 31.3±0.75 | 32.5±0.60 | 32.5±0.53 | 31.6±0.88 | 30.6±1.12 | 30.4±0.86 | 29.5±1.39 |
| ***p-value*** | 0.4432 | 0.1887 | 0.5253 | 0.8802 | 0.8881 | 0.7601 | 0.2976 | 0.4555 |
| **MAXVO_SIM_** | 27.6±1.31 | 29.1±1.70 | 29.9±1.82 | 30.9±1.23 | 29.7±1.66 | 29.5±1.72 | 28.0±1.40 | 28.4±2.30 |
| **MAXVO_ADJ2%_** | 27.8±0.98 | 29.1±1.31 | 29.9±1.49 | 31.1±0.86 | 33.5±1.16 | 29.2±1.18 | 28.0±1.02 | 28.4±1.62 |
| ***p-value*** | 0.4885 | 0.8629 | 0.9264 | 0.2993 | p<.0001 | 0.0863 | 0.9505 | 0.9870 |
| **CL_SIM_** | 28.1±1.27 | 28.8±1.15 | 29.1±1.22 | 29.4±1.25 | 28.6±1.24 | 28.3±1.32 | 26.1±1.32 | 27.5±2.04 |
| **CL_ADJ2%_** | 28.0±0.95 | 28.9±0.68 | 29.2±0.94 | 29.3±0.95 | 28.5±0.97 | 28.3±0.97 | 26.1±0.99 | 27.1±1.18 |
| ***p-value*** | 0.5449 | 0.8443 | 0.5440 | 0.3437 | 0.5432 | 0.9991 | 0.8674 | 0.2137 |

**Supplementary Table 14.** Simulated and adjusted means ± standard deviation of minimum cutaneous temperature of the dorsopalmar (DP) view from the Monte Carlo simulation. The horses were submitted to experimental induction of transient synovitis for both trials (C and MAXVO). C=control group; MAXVO received meloxicam (Maxicam Gel®), administered orally at 0.6 mg/kg daily three days before LPS injection; CL=contralateral limbs (negative control). The means and standard deviation of each treatment and moment were taken from the original data. The simulation was also adjusted for ± 2% accuracy.

| **Groups (*n*=100)** | **Timepoints (hours)** | | | | | | | |
| --- | --- | --- | --- | --- | --- | --- | --- | --- |
|  | **0** | **2** | **4** | **6** | **8** | **12** | **24** | **48** |
| **C_SIM_** | 27.1±1.56 | 30.6±1.01 | 31.8±1.30 | 31.8±0.90 | 31.1±1.36 | 29.4±1.61 | 29.2±1.06 | 29.03±1.61 |
| **C_ADJ2%_** | 26.9±1.11 | 30.6±0.80 | 35.3±0.88 | 31.8±0.74 | 31.1±0.90 | 29.0±1.12 | 29.1±0.72 | 28.93±1.1 |
| ***p-value*** | 0.3355 | 0.9641 | p<.0001 | 0.7879 | 0.8807 | 0.0511 | 0.5551 | 0.6127 |
| **MAXVO_SIM_** | 26.9±1.34 | 28.6±1.47 | 28.7±1.63 | 30.0±1.39 | 29.4±1.75 | 29.3±1.90 | 27.5±1.06 | 27.6±1.82 |
| **MAXVO_ADJ2%_** | 26.6±0.99 | 28.5±0.95 | 28.5±1.08 | 29.7±0.96 | 29.6±1.04 | 29.2±1.25 | 27.4±0.59 | 27.8±1.41 |
| ***p-value*** | 0.1383 | 0.4913 | 0.2253 | 0.1562 | 0.3656 | 0.7650 | 0.3106 | 0.5852 |
| **CL_SIM_** | 27.1±1.20 | 28.9±0.97 | 29.2±1.27 | 29.6±1.17 | 28.3±1.39 | 27.8±1.38 | 25.7±1.24 | 26.8±1.69 |
| **CL_ADJ2%_** | 27.1±0.87 | 28.7±0.71 | 29.2±0.83 | 29.4±0.84 | 28.4±1.02 | 28.1±0.96 | 25.8±0.98 | 26.9±1.07 |
| ***p-value*** | 0.9150 | 0.2806 | 0.7381 | 0.1443 | 0.5191 | 0.0866 | 0.4951 | 0.7328 |

**Supplementary Table 15.** Simulated and adjusted means ± standard deviation of minimum cutaneous temperature of palmarodorsal (PD) view from the Monte Carlo simulation. The horses were submitted to experimental induction of transient synovitis for both trials (C and MAXVO). C=control group; MAXVO received meloxicam (Maxicam Gel®), administered orally at 0.6 mg/kg daily three days before LPS injection; CL=contralateral limbs (negative control). The means and standard deviation of each treatment and moment were taken from the original data. The simulation was also adjusted for ± 2% accuracy.

| **Groups (*n*=100)** | **Timepoints (hours)** | | | | | | | |
| --- | --- | --- | --- | --- | --- | --- | --- | --- |
|  | **0** | **2** | **4** | **6** | **8** | **12** | **24** | **48** |
| **C_SIM_** | 28.1±1.20 | 29.5±1.43 | 31.8±1.17 | 31.2±1.43 | 31.7±1.04 | 28.2±1.20 | 28.6±1.07 | 29.2±1.18 |
| **C_ADJ2%_** | 28.0±0.96 | 29.5±0.86 | 31.9±0.96 | 31.2±0.95 | 31.9±0.64 | 28.3±0.69 | 28.6±0.81 | 29.2±0.81 |
| ***p-value*** | 0.7956 | 0.7936 | 0.5959 | 0.7818 | 0.0880 | 0.7291 | 0.8015 | 0.8699 |
| **MAXVO_SIM_** | 27.9±1.49 | 28.8±1.06 | 28.8±1.06 | 30.6±1.16 | 29.8±1.37 | 29.8±1.80 | 28.4±1.03 | 29.1±2.12 |
| **MAXVO_ADJ2%_** | 28.0±0.97 | 28.7±0.91 | 28.7±0.91 | 30.7±0.98 | 29.8±1.03 | 29.5±1.29 | 28.2±0.91 | 29.1±1.50 |
| ***p-value*** | 0.5306 | 0.5232 | 0.5232 | 0.7424 | 0.8996 | 0.1506 | 0.1357 | 0.9437 |
| **CL_SIM_** | 28.2±1.25 | 29.4±1.02 | 30.2±1.16 | 30.1±1.33 | 29.9±1.20 | 28.6±1.27 | 26.6±1.09 | 27.7±1.74 |
| **CL_ADJ2%_** | 28.1±0.83 | 29.4±0.71 | 30.2±0.82 | 30.1±0.73 | 29.8±0.82 | 28.5±0.87 | 26.7±0.85 | 27.6±1.13 |
| ***p-value*** | 0.4582 | 0.6055 | 0.7471 | 0.8463 | 0.3798 | 0.3663 | 0.5425 | 0.6006 |
